# Microemulsions of Nonionic Surfactant with Water and Various Homologous Esters: Preparation, Phase Transitions, Physical Property Measurements, and Application for Extraction of Tricyclic Antidepressant Drugs from Aqueous Media

**DOI:** 10.3390/nano13162311

**Published:** 2023-08-11

**Authors:** Radu C. Racovita, Maria D. Ciuca, Daniela Catana, Cezar Comanescu, Oana Ciocirlan

**Affiliations:** 1 Department of Inorganic Chemistry, Physical Chemistry and Electrochemistry, Faculty of Chemical Engineering and Biotechnologies, National University of Science and Technology Politehnica Bucharest, 1-7 Gh. Polizu St., District 1, 011061 Bucharest, Romania; maria_daniela.ciuca@upb.ro (M.D.C.); daniela.catana2911@upb.ro (D.C.); oana.ciocirlan@upb.ro (O.C.); 2 National Institute of Materials Physics, 405A Atomistilor St., 077125 Magurele, Romania; 3 Faculty of Physics, University of Bucharest, 405 Atomistilor St., 077125 Magurele, Romania

**Keywords:** microemulsions, esters, nonionic surfactant, co-surfactant, pseudo-ternary phase diagrams, phase transitions, electrical conductivity, dynamic viscosity, extraction, tricyclic antidepressant drugs

## Abstract

Microemulsions are nanocolloidal systems composed of water, an oil, and a surfactant, sometimes with an additional co-surfactant, which have found a wide range of practical applications, including the extractive removal of contaminants from polluted water. In this study, microemulsion systems, including a nonionic surfactant (Brij 30), water, and esters selected from two homologous series of C_1_–C_6_ alkyl acetates and ethyl C_1_–C_4_ carboxylates, respectively, were prepared by the surfactant titration method. Phase transitions leading to the formation of Winsor II and Winsor IV microemulsions were observed and phase diagrams were constructed. The dependences of phase transitions on the salinity and pH and the addition of isopropanol as a co-surfactant were also investigated. Some physical properties, namely density, refractive index, electrical conductivity, dynamic viscosity, and particle size, were measured for a selection of Winsor IV microemulsions, providing further insight into some other phase transitions occurring in the monophasic domains of phase diagrams. Finally, Winsor II microemulsions were tested as extraction solvents for the removal of four tricyclic antidepressant drugs from aqueous media. Propyl acetate/Brij 30/H_2_O microemulsions provided the best extraction yields (>90%), the highest Nernst distribution coefficients (~40–88), and a large volumetric ratio of almost 3 between the recovered purified water and the resulting microemulsion extract. Increasing the ionic strength (salinity) or the pH of the aqueous antidepressant solutions led to an improvement in extraction efficiencies, approaching 100%. These results could be extrapolated to other classes of pharmaceutical contaminants and suggest ester- and nonionic surfactant-based microemulsions are a promising tool for environmental remediation.

## 1. Introduction

Microemulsions have emerged over the past decades as a major research topic in colloid science. This is primarily due to their multiple areas of application, spanning fields like cosmetics [1,2,3], pharmaceuticals [4,5,6,7], agriculture [8,9], food [10,11,12], oil recovery [13,14,15], alternative fuels [16,17], catalysis [18,19,20], templated synthesis of nanoparticles [21,22,23,24,25,26], and environmental remediation [27,28,29,30,31].

**Figure 1:**
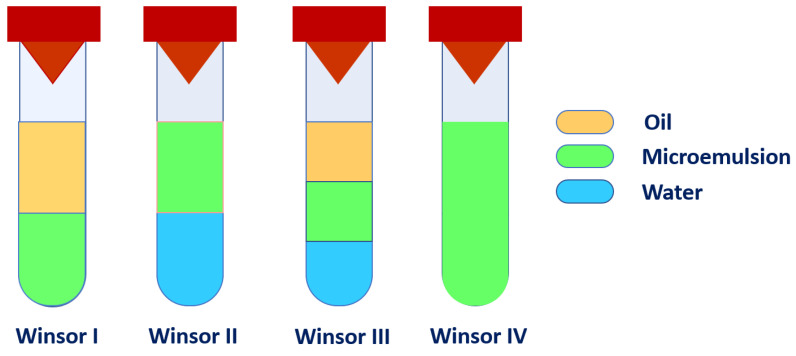
Microemulsion systems according to the Winsor classification (adapted from [7]).

Microemulsions are nanoscale colloidal systems with high thermodynamic stability, incorporating at least three components: water, oil, and a surfactant [7]. Often a fourth component is also included, known as a co-surfactant or co-solvent, which further enhances the stability of micelles dispersed in the continuous phase of the emulsion [26,32,33]. Another advantage of microemulsions is that they form spontaneously, as the surfactant (and co-surfactant, if present) lowers the free energy of the resulting nano-heterogeneous thermodynamic system. Unlike regular emulsions, microemulsions tend to be less viscous, visually transparent, and optically isotropic, in addition to having excellent long-term stability, which comes from the nanometric dimensions of their micelles, typically below 100 nm [6,10]. Similar to regular emulsions, microemulsions can be oil-in-water (O/W), where normal micelles made of oil with a surfactant coating are dispersed into a continuum of water, or water-in-oil (W/O), encompassing inverse micelles with water on the inside of a surfactant shell dispersed into the oil. The Winsor classification of microemulsions delineates four types of systems (Figure 1): Winsor I, consisting of an O/W microemulsion phase in equilibrium with a top oil phase; Winsor II, consisting of a W/O microemulsion phase in equilibrium with a bottom aqueous phase; Winsor III, representing an equilibrium between a top oil phase, a bottom aqueous phase, and a middle microemulsion phase, referred to as bicontinuous, which concentrates all the surfactant within intertwined domains of both W/O and O/W; and Winsor IV, which is a single phase that can be either O/W or W/O [7]. The particular type of Winsor system formed depends on the relative proportions of components, the choice of surfactant and/or co-surfactant, the presence of certain electrolytes, and environmental conditions [34].

As a result of their spontaneous formation and high thermodynamic stability, the fabrication of microemulsions is generally based on low-energy methods, typically the phase titration method and the phase inversion method [7]. The former consists of starting with binary mixtures with variable ratios of the two components to which the third component is slowly added (titrated) with minimal stirring. Most commonly, the oil and surfactant (plus co-surfactant, if it is the case) are mixed over a range of mass fractions and the water is titrated in small portions until the respective types of Winsor phases are observed visually [35,36,37]. It is, however, possible that the oil and water are mixed originally and the titration is performed using the surfactant [28,38]. Ternary (or pseudo-ternary if a co-surfactant is also involved) phase diagrams are then constructed, the various Winsor phase existence domains being delimited by frontiers (Winsor curves) given by the experimental compositions where the phase transitions have been observed [39,40]. Typically, the phase transitions observed with surfactant titration are either Winsor I→III→IV, Winsor II→III→IV, or Winsor III→IV [39]. Not all systems will show all of these transitions. For example, the Winsor III phase may not be observed or a system could form the monophasic Winsor IV from the first drop of added surfactant. The second fabrication method, the phase inversion technique, implies switching from O/W to W/O or vice versa by one of two options: either the addition of an excess amount of the dispersed phase at constant temperature (phase inversion composition, PIC) or a change in temperature at fixed composition (phase inversion temperature, PIT). The phase transition is triggered in PIC by the modification and fusion of micelle particles upon introduction of the excess component, while in PIT a decrease in surface tension upon cooling triggers the phase inversion [7].

The oil component of a microemulsion can be, in principle, any nonpolar, water-immiscible chemical compound. A wide range of substances have been used, depending on specific applications, ranging from hydrocarbons [41,42], supercritical CO_2_ [43], vegetable oils [11,17,44], essential oils [45], fatty acid methyl esters [46], and short chain esters [27,31].

A diversity of surfactant types can also be used for the preparation of microemulsions, ranging from cationic [47,48] to anionic [43,49] and nonionic [28,38,39]. There is a recent preference for the latter class, as these are more environmentally friendly than their ionic counterparts [39]. The most common co-surfactants reported are short-chain alcohols, like propanol, butanol, and pentanol [29,41,50,51], although other amphiphilic compound classes have been used occasionally [52].

In the field of environmental remediation, microemulsions have been employed mostly as low-volatility, environmentally benign extraction solvents for the decontamination of wastewater and recovery of valuable compounds [27,28,29,30,31,38,50]. In addition, recent work has shown that microemulsions can also serve as nanoreactors for the catalyst-free photodegradation of certain classes of environmental contaminants such as dyes [31]. Winsor I microemulsions make good extraction solvents for lipophilic compounds found in various oil fractions, leaving behind at equilibrium a clean upper oil phase, whereas Winsor II microemulsions are suitable for extracting hydrophilic compounds found in water, yielding a clean lower fraction of water (Figure 1). While Winsor I microemulsions have found their applicability, for example, as mobile phases in liquid chromatography [53,54], it is the latter type, Winsor II, that is of particular interest for the removal of contaminants from wastewater in environmental science. In particular, nonionic surfactant and ester-based Winsor II microemulsions have been recently applied successfully for the removal of diverse contaminants from real or simulated wastewater, including heavy metal ions, like cobalt [27,38,55], copper [27,29,55], nickel [39,50,55], chromium [55], iron, zinc, lead [29], and a series of lanthanides [56] as well as organic dyes such as crystal violet [27,30,31], methyl orange [30,31], and rhodamine B [27,30,31]. Although pharmaceutical drugs are another important class of micropollutants of wastewater and natural water, with severe impacts on both aquatic life and human health, like chronic toxicity, disruptions of the endocrine system, or increase of resistant bacterial strains [57,58], to our knowledge, there are no reports in the literature about the application of microemulsion extraction for the removal of such contaminants of emerging concern.

The present study was aimed at preparing and characterizing a range of microemulsion systems composed of several ester homologs, water, and a nonionic surfactant, investigating the phase transitions occurring in these systems and how they may be influenced by the salinity and pH of water or the addition of an alcohol co-surfactant, and finally applying them for the extractive separation of a selection of antidepressant drugs from their aqueous solutions, serving as simulated wastewater containing such compounds as model pharmaceutical contaminants.

## 2. Materials and Methods

### 2.1. Chemicals

Methyl acetate (MEAC, ≥99%), ethyl formate (ETFO, ≥98%), propyl acetate (PRAC, ≥99%), ethyl propionate (ETPR, ≥99%), ethyl butyrate (ETBU, ≥99%), and hexyl acetate (HEAC ≥ 99%) were purchased from Thermo Fisher Scientific. Ethyl acetate (ETAC, ≥99.8%) was from Honeywell, butyl acetate (BUAC, ≥99.5%) from Emsure and pentyl acetate (PEAC, ≥99%) from Supelco. Polyoxyethylene (4) lauryl ether (Brij 30, ≥99%, max. 1% H_2_O) was acquired from Acros Organics. Isopropanol (C3-ol, anh., ≥99.5%), chloroform (≥99.9%, with amylenes as stabilizer), sodium chloride (≥99%), sodium hydroxide (≥97%), phosphoric acid (85% in water), amitriptyline hydrochloride (AMI, ≥98%), doxepin hydrochloride (DOX, ≥98%, ~85% E isomer and 15% Z isomer), imipramine hydrochloride (IMI, ≥99%), and clomipramine hydrochloride (CLO, ≥98%) were from Sigma Aldrich. Mirtazapine (MIR, ≥98%) was purchased from Thermo Fisher Scientific. Helium 6.0 (≥99.9999%) was purchased from Linde GmbH. Doubly distilled water from an in-house distillation setup was used in all experiments. Brij 30 was dried at 105 °C overnight before use. All other reagents were used as received.

### 2.2. Preparation of Microemulsions by Surfactant Titration

The surfactant titration method was used in all experiments [28]. The temperature was always 25 °C. A series of test tubes were first loaded with mixtures of ester and water (totaling 1 mL in all experiments) and spanning the full range of water mass fractions from 0.95 to 0.05, in 0.05 decrements, to enable the construction of full ternary diagrams. Water and ester densities at 25 °C were used to convert between mass and volume. The aforementioned water mass fractions correspond to volumetric ratios R between water and ester from a high 19.0 down to a low 0.05. The dry nonionic surfactant, Brij 30, was added drop-wise (titrated) using Pasteur pipettes into the water and ester mixtures, with gentle manual shaking after each drop, until two clear liquid layers were obtained, marking the formation of a Winsor II microemulsion. At this point, the test tubes were weighed on an analytical balance Kern ABD 200-4 (KERN GmbH, Bensheim, Germany), with a precision of ±0.0001 g, and the mass of Brij 30 added was determined by difference. Titration with Brij 30 was continued until the whole mixture became a clear, single phase, corresponding to the Winsor IV microemulsion. The total mass of Brij 30 added was also determined by weighing by difference.

In the case of pseudo-ternary systems, the same procedure was applied with the only difference that water was replaced by mixtures of isopropanol and water with the mentioned volumetric ratios, aqueous NaCl solutions with stated molar concentrations, phosphate buffer solutions with indicated pH values. Buffers were prepared by slowly adding aqueous NaOH to aqueous H_3_PO_4_ until attaining the desired pH, measured with a stationary pH meter pH50+ DHS (Giorgio Bormac, Carpi, Italy).

### 2.3. Construction of Ternary and Pseudo-Ternary Phase Diagrams

Phase diagrams of ternary and pseudo-ternary systems [39,59] were constructed using Origin Pro 8.5 software (Origin Lab Corp., Northampton MA, USA) by plotting the data points corresponding to the compositions (mass fractions) of mixtures that mark the beginning of the Winsor II and Winsor IV domains, respectively. Mass fractions of the three components were computed with the formula:

(1) $$w_{i}=\frac{m_{i}}{\sum_{j=1}^{3}m_{j}}$$

where: *w_i_*—the mass fraction of component *i*;

*m_i_*—the mass of component *i*;

*m_j_*—the mass of component *j*.

### 2.4. Physical Property Measurements

The densities of the microemulsions were measured with an automated 4500 DMA densimeter (Anton Paar, Graz, Austria) based on the vibrating tube method with a precision of ±0.00005 g/cm^3^. The DMA cell was calibrated with dry air and doubly distilled water, while a built-in Peltier thermostat maintained temperature at 25 °C.

Refractive indices were measured at 25 °C, using the sodium D line, with a thermostated Abbé refractometer, having a precision of ±0.0001.

Electrical conductivities were recorded at 25 °C with a conductivity probe, part of the Cobra 3 Chem-Unit (PHYWE Systeme GmbH & Co. KG, Göttingen, Germany). Before the measurement, the conductivity cell was calibrated with two certified solutions (84 and 1413 µS/cm). The uncertainty of the conductivity measurements was ±0.5%.

Viscosities were determined with a rolling-ball AMVn microviscometer (Anton Paar, Graz, Austria), equipped with a built-in Peltier thermostat for temperature control. All measurements were performed at an inclination angle of 70° using suitable calibrated glass capillary tubes (1.8 and 3.0 mm). The relative uncertainty of viscosity measurements was less than ±2%. The Newtonian behavior of the systems was verified using different angles of inclination (70° and 30°), when identical viscosity values were obtained, within experimental errors.

The average size of micelle particles and their polydispersity index (PDI) was determined using the Stokes-Einstein equation from dynamic light scattering (DLS) measurements of undiluted microemulsions using a Zetasizer NanoZS apparatus (Malvern Instruments Inc., Malvern, UK). Measurement parameters were as follows: solid-state laser source, measurement angle of 173°, and temperature set at 25 °C.

All measurements were performed on three independent replicates and results are reported as averages.

### 2.5. Extraction of Tricyclic Antidepressants (TCAs) into Microemulsions

In all extraction experiments, the Winsor II microemulsion was generated in situ.

The following general protocol was applied: to 9 mL of TCA solution (prepared by dissolving the necessary amount of solid TCA in water, NaCl 0.5 M, or phosphate buffer) 1 mL of ester was added as an oil component. The mixture was placed on the analytical balance and Brij 30 was added drop-wise with occasional gentle stirring until the necessary mass for forming a Winsor II system was reached for the respective ester (according to its determined phase diagram). The resulting mixture was drawn into a 20-mL syringe, which was sealed and left overnight for the two Winsor II phases (W/O microemulsion-µE and excess water-W) to separate and the TCA to partition and equilibrate between the phases.

After equilibration, the volumes of the two separated phases were measured, *V_µE_* and *V_W_*, respectively. Next, 1 mL from the bottom aqueous phase was evacuated through the syringe needle into a clean glass vial for the purpose of determining the concentration of the remaining *TCA* in the aqueous phase and *C_TCA,aq_* using gas chromatography mass spectrometry, GC-MS (see Section 2.6). The concentration of *TCA* extracted into the microemulsion (*µE*) phase, *C_TCA,µE_*, was determined by mass balance, i.e., by subtracting the mass of *TCA* found in the *V_W_* mL of aqueous phase after partitioning from the initial total mass of *TCA* found in the original 9 mL of solution and dividing the resulting difference by *V_µE_*.

The extraction yield was calculated with the formula:

(2) $$\eta \left[\%\right]=\frac{C_{TCA}^{0}\cdot V_{sol}^{0}-C_{TCA,aq}\cdot V_{W}}{C_{TCA}^{0}\cdot V_{sol}^{0}}\cdot 100$$

where: *η*—the extraction yield, in %;

*C*^0^*_TCA_*—the initial concentration of *TCA* before extraction, in mg/L;

*V*^0^*_sol_*—the initial volume of the solution of *TCA* subjected to extraction, in mL;

*C_TCA,aq_*—the final concentration of *TCA* remaining in water after extraction, in mg/L;

*V_W_*—the final volume of water after phase partitioning, in mL.

### 2.6. Determination of Residual Concentrations of TCAs in Water after Extraction

The 1 mL of residual water collected after extraction was evaporated to dryness at 70 °C. The resulting residue was taken up with chloroform into a 1.5-mL GC vial to which a known amount of an internal standard solution (mirtazapine in chloroform) was added. All residual TCAs were quantified by comparison of their peak areas against those of the internal standard with a known concentration. For accurate quantification, calibration curves were prepared in advance for each TCA mixed with the internal standard using a series of TCA solutions with exact concentrations.

Chromatographic analyses were carried out using a Thermo Scientific Focus GC coupled with a Polaris Q ion trap MS detector. The splitless injection mode at an inlet temperature of 250 °C was employed, the GC-MS being equipped with a TriPlus Autosampler. The capillary column was TR-5MS from Thermo Fisher Scientific (length = 30 m; internal diameter = 0.25 mm; film thickness = 0.25 µm). Helium served as the carrier gas, at a constant flow rate of 1 mL/min. The ion source temperature was set to 250 °C, and the MS transfer line was set to a slightly higher temperature of 280 °C. The oven temperature program was as follows: start at 60 °C and hold for 3 min; ramp up to 300 °C at a rate of 15 °C/min; hold at 300 °C for 4 min. For enhancing sensitivity, all measurements were performed in single-ion monitoring (SIM) mode, as per other studies [60,61,62,63,64,65]. The specific product ions selected for monitoring each of the four TCA analytes and the internal standard were, in the order of elution: *m*/*z* 58 for AMI; *m*/*z* 58 for DOX; *m*/*z* 234 for IMI; *m*/*z* 195 for MIR; and *m*/*z* 268 for CLO. These ions were selected because they produced the most intense peaks in the mass spectra of the respective compounds, thus maximizing detection sensitivity.

## 3. Results and Discussion

### 3.1. Ternary Phase Diagrams of Prepared Microemulsion Systems

#### 3.1.1. Changes in Phase Diagrams with the Homology of the Alkyl Part C_x_ (x = 1–6) of Alkyl Acetates (C_x_ + C_2_) Used as Oil Component of W/O/Brij 30 Microemulsion Systems

**Figure 2:**
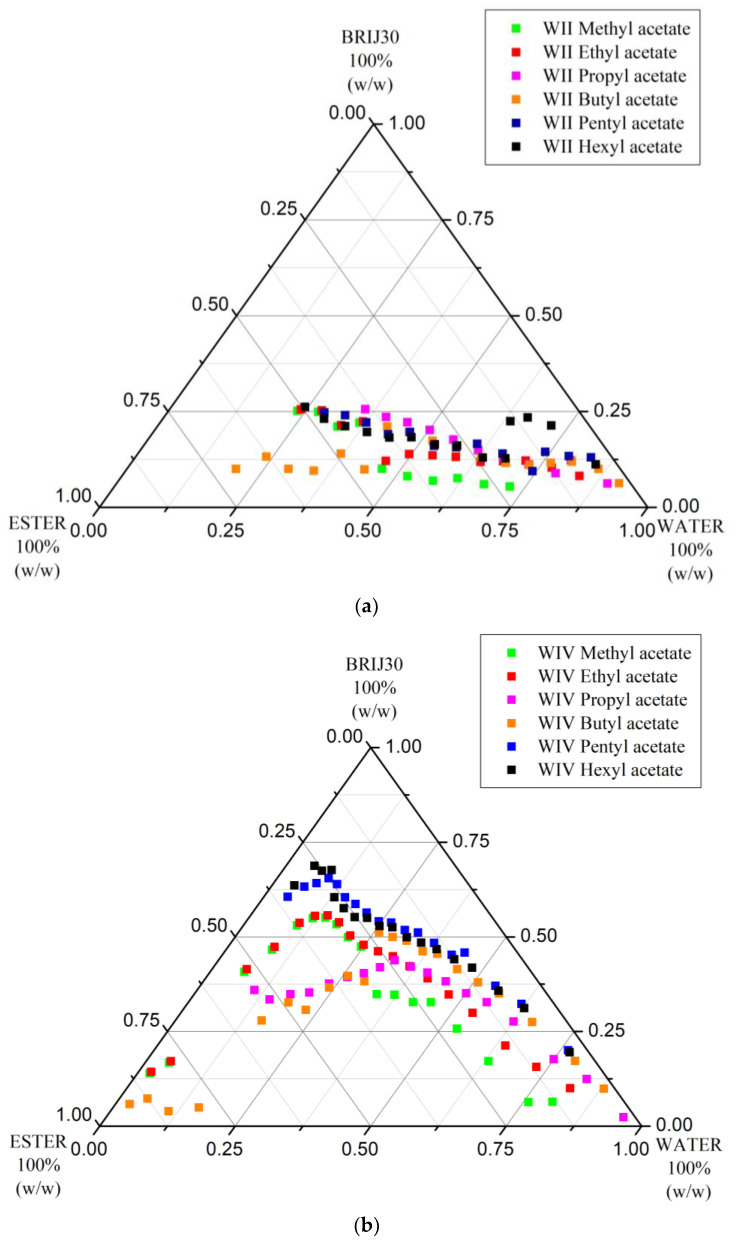
(**a**) Winsor II curves of ternary systems incorporating water, Brij 30, and alkyl (C_1_–C_6_) acetates (t = 25 °C). (**b**) Winsor IV curves of ternary systems incorporating water, Brij 30, and alkyl (C_1_–C_6_) acetates (t = 25 °C).

Figure 2a,b show comparatively the Winsor II curves (i.e., the lower borders of Winsor II domains, where just enough surfactant has been added to the initial ester and water mixtures to obtain a Winsor II system) and the Winsor IV curves (i.e., the lower borders of Winsor IV domains, where the amount of surfactant added is sufficient to create a single phase in the whole system) for six ternary systems employing different acetate homologs. These curves essentially make up the ternary phase diagrams. In these diagrams, the mass fractions of the three components (water, ester, and surfactant) are plotted on the three sides of the Gibbs-Roozeboom equilateral triangle [39]. Between the bottom side of the triangle and the Winsor II curve, each system is inhomogeneous and does not constitute a microemulsion. Between its Winsor II and Winsor IV curves, each ternary mixture forms a Winsor II system, made of a clear, transparent W/O microemulsion layer on top of the excess water layer. Above their corresponding Winsor IV curve (the binodal curve), all ternary systems become single-phase systems [59], i.e., Winsor IV microemulsions.

Using Origin Pro 8.5 software, the areas below each curve in the diagrams were computed and the relative areas are reported as % of the total triangle area in Table 1.

As can be seen from Figure 2a and Table 1, the lowest-lying Winsor II curve corresponds to the system with MEAC (C_2_ acetate) and the highest to the one with HEAC (C_6_ acetate). This means that, in general, the smallest amount of Brij 30 surfactant that needs to be titrated into the initial ester and water mixtures to reach a Winsor II system is minimal in the case of the shortest alkyl acetate and maximized when the longest homolog is used as the oil component. Since the formation of the clear W/O Winsor II microemulsion implies miscibilization of the ester and water with the aid of the added surfactant, it is not surprising that the longer and more hydrophobic esters (BUAC, PEAC, and HEAC) require higher amounts for nonionic surfactant than the shorter ones (MEAC, ETAC, and PRAC).

The Winsor II microemulsions can serve as extraction systems for various analytes from aqueous media, for example, the removal of contaminants from wastewater or other contaminated water [27,28,29,38]. Therefore, a larger Winsor II domain area in the phase diagram would be desirable for such purposes. However, the data in the third column of Table 1 should be judged only when taking into account the corresponding data in the other columns. It would be desirable that the enlargement of the Winsor II domain is the result of a lower Winsor II curve rather than an upper Winsor IV curve (marked by a reduction of the Winsor IV domain area in Table 1), the latter corresponding to higher consumption of Brij 30 surfactant, which would imply a higher cost. Clearly, although BUAC yielded the largest Winsor II domain, this domain is delimited by high-lying Winsor II and Winsor IV curves, i.e., requires high amounts of surfactant to form. The same is true for the other longer chain alkyl esters, PEAC and HEAC. ETAC has fairly high-lying domain frontiers also, such that MEAC appears to yield the largest Winsor II domain encompassed by the lowest-lying Winsor II and Winsor IV curves, followed by PRAC. However, a closer look at Figure 2a reveals that, due to the higher miscibility of MEAC with water, Winsor II phases are not obtained at high water content (bottom right corner of the phase diagram). This region is of particular interest for water decontamination because the small amount of ester oil leads to small amounts of W/O Winsor II microemulsions that extract and concentrate the contaminants, leaving behind at equilibrium very large amounts of decontaminated water, as desired. Furthermore, the high volatility of MEAC (b.p. 57 °C [66]) makes it an inconvenient ester for practical purposes. On the other hand, these inconveniences no longer exist in the case of PRAC (b.p. 102 °C [67]), which would thus be a more suitable ester from a practical standpoint.

#### 3.1.2. Changes in Phase Diagrams with the Homology of the Acyl Part C_y_ (y = 1–4) of Ethyl Carboxylates (C_x_ + C_2_) Used as Oil Component of W/O/Brij 30 Microemulsion Systems

**Figure 3:**
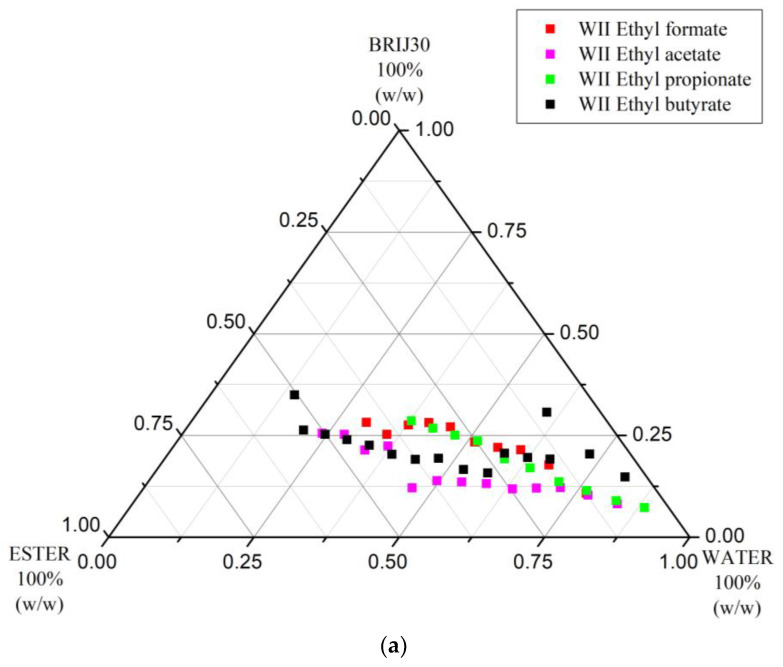

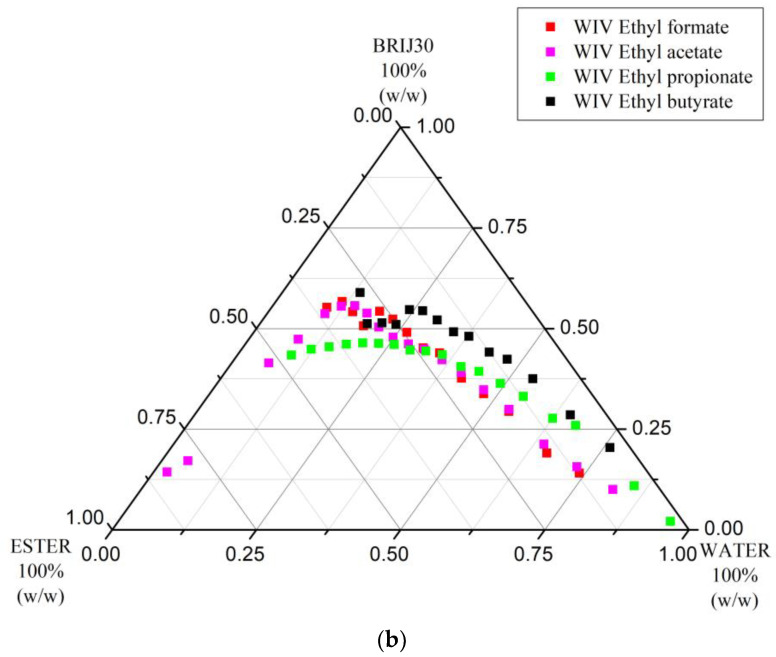
(**a**) Winsor II curves of ternary systems incorporating water, Brij 30, and ethyl carboxylates (C_1_–C_4_) (t = 25 °C). (**b**) Winsor IV curves of ternary systems incorporating water, Brij 30, and ethyl carboxylates (C_1_–C_4_) (t = 25 °C).

Figure 3a,b show comparatively the Winsor II and Winsor IV curves, respectively, for four ternary systems employing different ethyl carboxylate homologs, while Table 2 summarizes the % areas of the domains delimited by these curves in the phase diagrams. From the data, it can easily be seen that, although the Winsor II domain for the ternary system with ETAC is the largest, both the Winsor II and Winsor IV curves are uplifted in comparison with those of ETFO and ETPR; therefore, surfactant demand is higher. ETBU has both the narrowest Winsor II area and the highest surfactant consumptions for achieving either Winsor II or IV phases. Similar to its metamer MEAC, ETFO did not allow the formation of Winsor II phases at high W/O ratios (Figure 3a, bottom right corner), its volatility is inconveniently high (b.p. 54 °C [68]) and the Winsor II domain is the smallest. Thus, the metamer of PRAC, ETPR (b.p. 99 °C [69]), is most convenient for Winsor II extractions in this homologous series.

Based on the results above, all further work on establishing and characterizing phase transitions in these microemulsion systems was focused exclusively on PRAC and ETPR as oil components.

Ternary microemulsion-forming systems based on water, Brij 30, and ETAC have been prepared before and are described in the literature [28,30,31,38,39,50]. Mihaly et al. were the first to publish the phase diagram of this system at 25 °C [50]. Our phase diagram is nearly identical to theirs within experimental errors. Fleancu et al. went further and studied how the phase diagram changes with changes in temperature and noted that only at temperatures below 15 °C are Winsor I phases observed, while temperatures higher than 40 °C caused an enlargement of Winsor II phase domains [39]. This explains why we did not observe any Winsor I domains, as our working temperature exceeded the phase inversion temperature of 15 °C.

Similarly, the system with BUAC as an oil component has also been reported several times [27,28,29,38,55], although always as an extraction system for either metallic ions or dyes from contaminated water. There is no complete phase diagram available in the literature for this ternary system. Cadar et al. reported a pseudo-ternary phase diagram where acetone was added to water as a fourth component and the aqueous phase was in fact a solution of [Co(SCN)_4_]^2−^ ions [28,38]. While their Winsor II curve is very close to ours, the Winsor IV curve is much lower, likely because acetone increases the miscibility of the phases.

ETBU has also been reported as the oil component of some microemulsions for various drug delivery systems, albeit with other nonionic surfactants, such as Brij 97 and Tween 80 [70] or polyoxyethylene-10-dodecyl ether [71]. However, phase diagrams have not been reported. All other ester-based ternary systems presented herein have not been described in the literature to the best of our knowledge.

### 3.2. Effect of Co-Surfactant, Salinity, and pH on the Phase Transitions of the Microemulsion Systems

#### 3.2.1. Effect of Addition of Different Amounts of Isopropanol as a Co-Surfactant

The changes in phase boundaries caused by the addition of different proportions of an alcohol co-surfactant/co-solvent, i.e., isopropanol (C3-ol), to the initial aqueous component of the mixture were investigated. Due to its amphiphilic nature, the co-surfactant can get incorporated into the surfactant layer at the surface of micelles, with its hydrophobic part aligned to the hydrocarbon chain of the surfactant and its polar functional group with the polar part of the surfactant and in the vicinity of water molecules [51]. The presence of the co-surfactant at this interface typically causes a change in the interfacial energy and may affect phase transitions, phase boundary curvatures, and locations, as a consequence of modification of volumetric ratios between water/surfactant or oil/surfactant [50].

**Figure 4:**
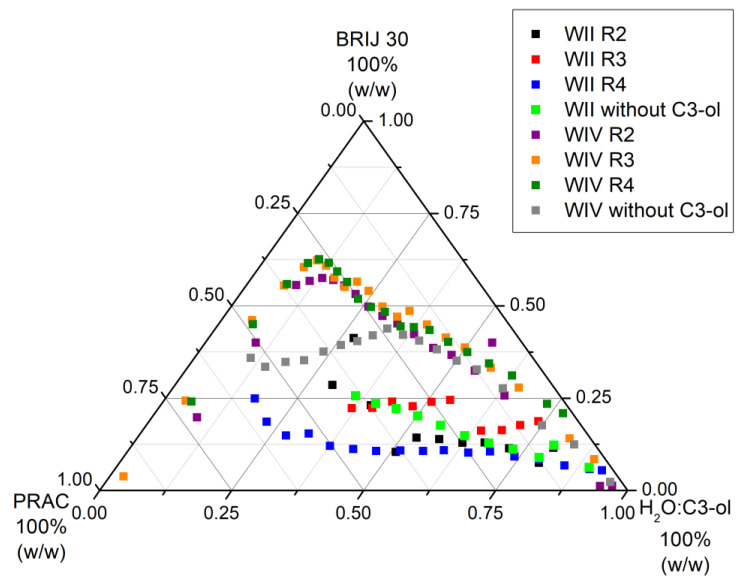
Winsor II and IV curves of pseudo-ternary systems made of water (H_2_O) with and without isopropanol (C3-ol), Brij 30, and PRAC (R is the volumetric ratio H_2_O: C3-ol; t = 25 °C).

**Figure 5:**
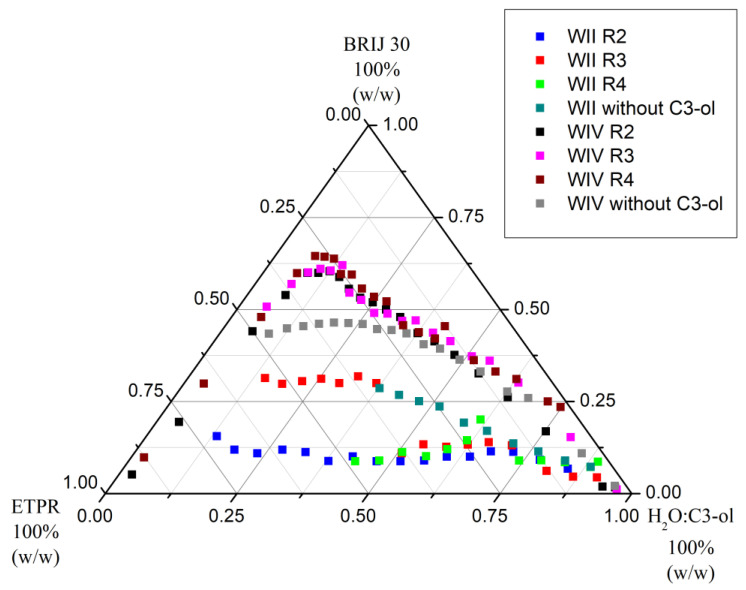
Winsor II and IV curves of pseudo-ternary systems made of water (H_2_O) with and without isopropanol (C3-ol), Brij 30, and ETPR (R is the volumetric ratio H_2_O: C3-ol; t = 25 °C).

Figure 4 and Figure 5 depict the shifts of Winsor II and Winsor IV curves of the pseudo-ternary systems with PRAC and ETPR, respectively, as oil components when C3-ol co-solvent was added to water at initial volumetric ratios H_2_O/C3-ol, R, equal to 2, 3, and 4, as shown in the figure legends.

Table 3 and Table 4 include the relative areas (% of total area) of the various domains delimited by the phase boundaries in Figure 4 and Figure 5 for the pseudo-ternary systems with PRAC and ETPR, respectively.

From the data in Table 3 regarding the systems with PRAC as ester oil, it is immediately apparent that the addition of C3-ol favored phase separation towards the formation of Winsor II microemulsions, given that the Winsor II domains are larger for ratios R equal to 3 and 4. Especially for R = 4, there is a noticeable extension towards the left of the diagram of the Winsor II curve (Figure 4), meaning that this level of added co-surfactant makes it possible to attain Winsor II systems with high ester content, but this is not necessarily a desirable feature for water decontamination because a larger amount of the water would get incorporated into the W/O microemulsion along with the contaminants and less purified water would result. In contrast, for R = 2, the Winsor II domain decreased in comparison with the ternary system lacking a co-surfactant, with too much alcohol making it difficult for the aqueous and oil phases to separate. This type of differential phase behavior, where, up to a certain amount of added co-surfactant, the Winsor II domain widens and then it narrows down past that amount, has been reported before for a related system, water and isopropanol/Brij 30/pine oil [45].

In the case of systems with ETPR (Table 4), the addition of C3-ol extended Winsor II domains in all cases, the area is greater the lesser the proportion of added C3-ol (the higher the R ratio). Nonetheless, for both PRAC and ETPR-based systems, the Winsor II not only extended farther left on the phase diagrams but also shifted higher up (Figure 4 and Figure 5 and the second column of Table 3 and Table 4). This effect corresponds to a higher consumption of Brij 30 to reach Winsor II phases, which is undesirable in practice. In fact, when inspecting the last columns of Table 3 and Table 4, it becomes clear that the wider Winsor II domains are mostly the result of narrower Winsor IV domains (uplifting of Winsor IV curves) combined with the occasional extension to the left of Winsor II curves, none of which is consistent with the generation of maximum purified water with minimum surfactant and ester consumption.

The utilization of isopropanol as a co-surfactant does not offer any benefits for compositions characterized by high water content and minimal ester oil presence (located in the lower right corners of the phase diagrams depicted in Figure 4 and Figure 5), which are particularly relevant in the context of contaminated water remediation.

#### 3.2.2. Effect of Salinity (NaCl Content) of the Water Component

**Figure 6:**
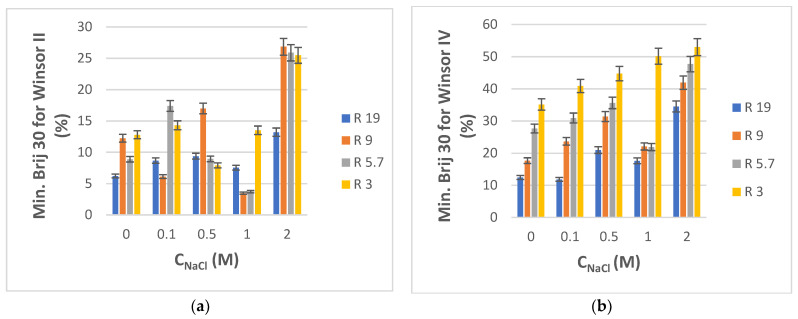
(**a**) Minimum weight % of Brij 30 needed to obtain Winsor II microemulsion for H_2_O + NaCl/Brij 30/PRAC systems as a function of NaCl concentration in water. R is the volumetric ratio H_2_O + NaCl/PRAC. t = 25 °C. (**b**) Minimum weight % of Brij 30 needed to obtain Winsor IV microemulsion for H_2_O + NaCl/Brij 30/PRAC systems as a function of NaCl concentration in water. R is the volumetric ratio H_2_O + NaCl/PRAC. t = 25 °C.

**Figure 7:**
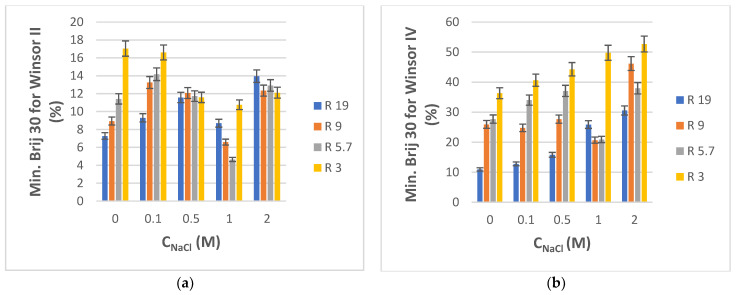
(**a**) Minimum weight % of Brij 30 needed to obtain Winsor II microemulsion for H_2_O + NaCl/Brij 30/ETPR systems as a function of NaCl concentration in water. R is the volumetric ratio H_2_O + NaCl/ETPR. t = 25 °C. (**b**) Minimum weight % of Brij 30 needed to obtain Winsor IV microemulsion for H_2_O + NaCl/Brij 30/ETPR systems as a function of NaCl concentration in water. R is the volumetric ratio H_2_O + NaCl/ETPR. t = 25 °C.

Although laboratory research studies are typically carried out with doubly distilled or other types of high-purity water, environmental aqueous samples are almost always contaminated not just by a certain class of particular contaminants, but also by many other components that make up a very complex matrix. Inorganic salts are ubiquitous, so, in order to model how their presence might cause changes in phase transitions, we investigated the effect of water salinity by incorporating NaCl of different concentrations into the starting aqueous phase. For this purpose, only four compositions from the water-rich end of the phase diagrams of both PRAC and ETPR-based systems were chosen, namely those corresponding to volumetric water/ester ratios R equal to 19, 9, 5.7, and 3. The salinity of the water was varied also: 0.1, 0.5, 1, and 2 M NaCl. The minimal amounts of Brij 30 surfactant (reported as % weight of total pseudo-ternary system) that needed to be titrated into the NaCl solution and ester mixtures in order to obtain Winsor II and Winsor IV phases, respectively, are plotted in Figure 6a,b for PRAC- and Figure 7a,b for ETPR-based systems.

Overall, the minimum consumption of Brij 30 to attain Winsor II and especially Winsor IV microemulsions increases with an increase in water salinity (Figure 6 and Figure 7). This higher demand for surfactant for the formation of microemulsion phases in electrolyte media has been reported before and explained as a consequence of the decrease of the effective volume of polar heads of surfactant in the presence of inorganic ions and thus of the number of hydrogen bonds between surfactant and water, which would otherwise stabilize the microemulsion [72,73]. The increased demand of Brij 30 with salinity is less pronounced and more irregular in the case of Winsor II systems (Figure 6a or Figure 7a) than with Winsor IV systems where it is more consistent and proportional with NaCl concentration (Figure 6b or Figure 7b). Only in the case of PRAC and when the salt content was highest (2 M), there was a substantial increase in Brij 30 consumption (Figure 6a). For NaCl concentrations up to 1 M, there is little, if any, increase in the Brij 30 needed to produce Winsor II microemulsion in comparison with systems lacking salt (Figure 6a or Figure 7a).

#### 3.2.3. Effect of the pH of Water

**Figure 8:**
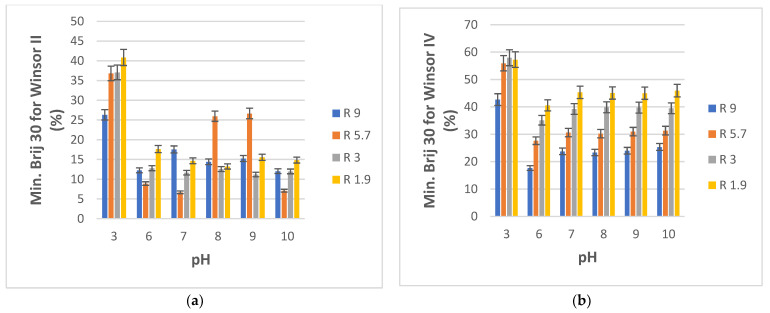
(**a**) Minimum weight % of Brij 30 needed to obtain Winsor II microemulsion for aqueous phosphate buffer/Brij 30/PRAC systems as a function of buffer pH. R is the volumetric ratio buffer/PRAC. t = 25 °C. (**b**) Minimum weight % of Brij 30 needed to obtain Winsor II microemulsion for aqueous phosphate buffer/Brij 30/PRAC systems as a function of buffer pH. R is the volumetric ratio buffer/PRAC. t = 25 °C.

**Figure 9:**
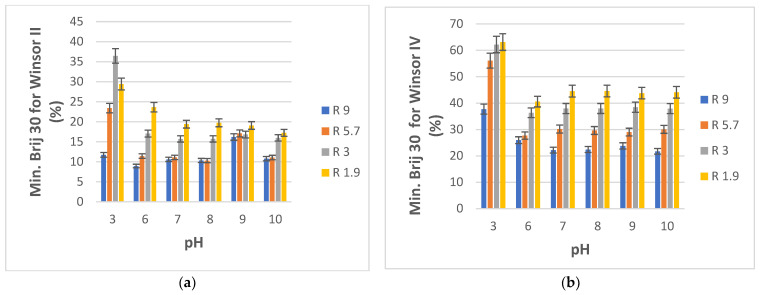
(**a**) Minimum weight % of Brij 30 needed to obtain Winsor II microemulsion for aqueous phosphate buffer/Brij 30/ETPR systems as a function of buffer pH. R is the volumetric ratio buffer/ETPR. t = 25 °C. (**b**) Minimum weight % of Brij 30 needed to obtain Winsor IV microemulsion for aqueous phosphate buffer/Brij 30/ETPR systems as a function of buffer pH. R is the volumetric ratio buffer/ETPR. t = 25 °C.

The pH of the aqueous component was varied using buffer systems in order to investigate how the formation of Winsor II and IV microemulsions may be influenced. This influence presents interest also because wastewater and natural effluents are likely to have a variety of pH values as a result of the various contaminants they may contain. Once again, only four compositions closer to the water-rich ends of the PRAC and ERPR-system diagrams were selected, specifically those corresponding to volumetric water/ester ratios R equal to 9, 5.7, 3, and 1.9. The pH of water varied from a low of 3 to a high of 10. Our in-house doubly distilled water had a pH of 6. The desired aqueous phase pH values were attained using phosphate buffers, by slowly mixing aqueous solutions of NaOH and H_3_PO_4_ until reaching the desired pH as indicated by a pH meter. The minimal amounts of Brij 30 surfactant (reported as % weight of total pseudo-ternary system) that needed to be added to the aqueous buffer and ester mixtures as a function of pH in order to obtain Winsor II and Winsor IV phases, respectively, are plotted in Figure 8a,b for PRAC-based systems and in Figure 9a,b for ETPR-based systems.

What stands out from Figure 8a,b and Figure 9a,b is a substantially higher demand for surfactant for both systems (PRAC and ETPR) in order to obtain Winsor II as well as Winsor IV microemulsions when the pH is 3 as compared to the usual pH 6 of water. Thus, highly acidic aqueous phases make it more difficult to obtain ester-based microemulsions. For the PRAC system, only for R = 5.7, there was a rather important increase in Brij 30 consumption to achieve a Winsor II microemulsion at pH values of 8 and 9 vs. the usual pH of 6. On the other hand, there appears to be no substantial change in surfactant demand for obtaining Winsor IV microemulsions for all pHs from 6 to 10, irrespective of aqueous/oil phase initial ratio R. Similarly, for ETPR, neutral and basic pH values produce no change in Brij 30 demand for attaining a Winsor IV microemulsion as compared to the slightly acidic pH 6, while for attaining a Winsor II microemulsion there is only a somewhat sizeable increase for a pH of 9 in the case of R = 9 and R = 5.7. In general, basic pH values of the water phase do not influence microemulsion phase formation.

### 3.3. Characterization of a Selection of Microemulsions

#### 3.3.1. Densities of Microemulsions

Density is an important physical property of microemulsions but was also determined here as prerequisite input data for the measurement of viscosities using the automated AMVn viscometer, viscosities that can provide further insight into some phase transitions possibly occurring [74,75]. Densities of the single components were measured first at 25 °C for reference and found to be as follows (in g/cm^3^): 0.997 for water, 0.95 for Brij 30, 0.887 for both PRAC and ETPR, and 0.786 for C3-ol.

**Figure 10:**
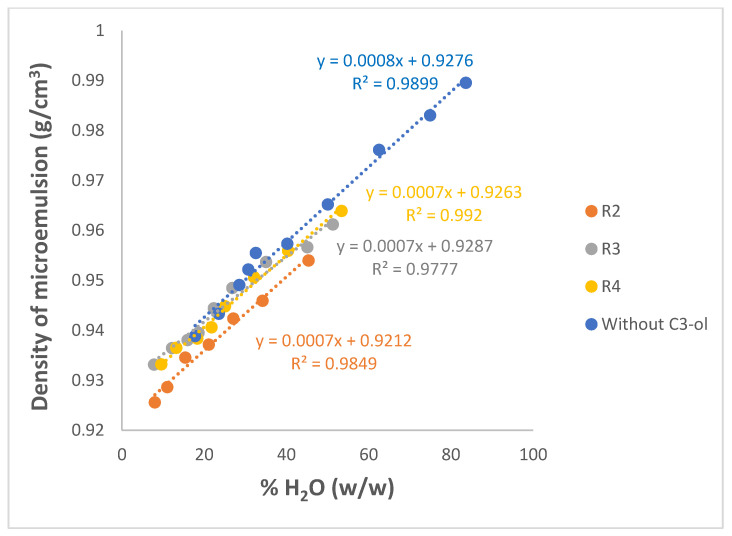
Variation of densities as a function of the weight % of H_2_O incorporated for Winsor IV microemulsion systems H_2_O/Brij 30/PRAC without and with different proportions of added C3-ol co-surfactant. R is the volumetric ratio H_2_O/C3-ol. t = 25 °C.

Figure 10 and Figure S1 show the variation of measured densities of Winsor IV microemulsions with the weight % of the water component of the microemulsions, for the pseudo-ternary systems incorporating PRAC and those incorporating ETPR, respectively. Both systems without co-surfactant C3-ol and those with C3-ol were measured, R values shown being once again the volumetric ratios H_2_O/C3-ol, ranging from 2 to 3 to 4.

Figure 10 and Figure S1 show consistently a linear increase of densities with increasing water content, irrespective of the ester used as an oil component or of the proportion of C3-ol added. This suggests, on the one hand, that the concentration of the densest component, i.e., water, is a determinant for the overall density and, on the other hand, that a simple density measurement could be used to interpolate the water % of the microemulsions based on the linear equations shown above. As expected, the more of the C3-ol was added as a co-solvent to replace part of the water (lower R), the smaller the densities obtained of the respective microemulsions as a result of the much smaller density of C3-ol. Working with benzene, water, Tween 20, and heptanol microemulsions, Mehta and Bala also observed that, irrespective of the relative composition, densities always remained >1 g/cm^3^, dominated by their only component with a density exceeding 1 g/cm^3^, i.e., the Tween 20 surfactant [75].

Densities of the microemulsions were also estimated by using the following mixing rule:

(3) $$\rho_{calc}=\sum_{i}\frac{\%\;\mathrm{comp}.\;i}{100}\cdot \rho_{i}$$

in which: *ρ_calc_*—the calculated density of microemulsion;

% comp. *i*—the mass percent of component *i* (water, ester, Brij 30, and C3-ol);

*ρ_i_*—the density of component *i.*

The results of the calculations are shown in Supplementary Tables S1 and S2 for PRAC and ETPR, respectively. From the data presented in these tables, it can be concluded that such estimated densities are fairly close to the real, experimental values, showing deviations well below 5%.

#### 3.3.2. Refractive Indices of Microemulsions

**Figure 11:**
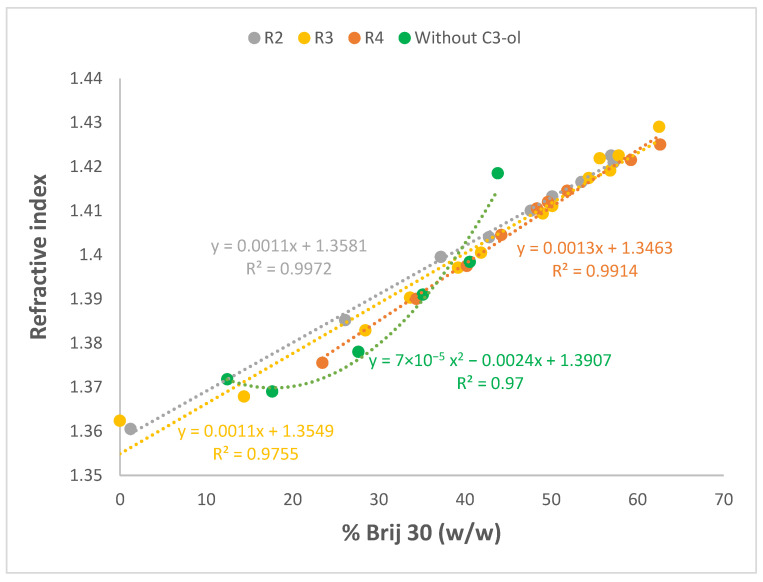
Variation of refractive indices as a function of the weight % of H_2_O incorporated for Winsor IV microemulsion systems H_2_O/Brij 30/PRAC without and with different proportions of added C3-ol co-surfactant. R is the volumetric ratio H_2_O/C3-ol. t = 25 °C.

For the same selection of microemulsions, refractive indices were measured at 25 °C, and the values obtained are plotted in Figure 11 and Figure S2 for PRAC and ETPR systems, respectively, with and without a co-surfactant. Reference refractive indices for the single components were also recorded at 25 °C: 1.3352 for water, 1.3776 for C3-ol, 1.3845 for ETPR, 1.3848 for PRAC, and 1.4525 for Brij 30, respectively.

For all pseudo-ternary systems incorporating C3-ol co-surfactant, refractive indices increased linearly with the weight % of the Brij 30 surfactant, while for the ternary systems lacking C3-ol, there was also an increasing trend, albeit not linear. The data suggest that the refractive indices of microemulsions are determined primarily by the component with the highest refractive index, i.e., Brij 30, and thus the experimentally determined refractive index could serve as a direct indicator of the concentration of this component on the basis of the curves above (Figure 11 and Figure S2).

The range of refractive indices obtained here, 1.36–1.43, is quite typical of microemulsions incorporating water, esters, nonionic surfactants, and alcohol co-surfactants. Working with isopropyl palmitate as the oil, water, and a variety of Tween surfactants together with 1-butanol as the co-surfactant, Basheer et al. obtained refractive indices ranging from 1.39 to 1.44 [51]. Hermansky and Mackay showed that, even when the oil was not an ester but an alkane, hexadecane, in combination with Brij 96 and 1-butanol, refractive indices remained similar, between 1.34 and 1.44 [76].

#### 3.3.3. Electrical Conductivities of Microemulsions

Electrical conductivity measurements have been used in past studies as experimental evidence for delineating the specific type of Winsor IV microemulsions: water-in-oil (W/O), oil-in-water (O/W), or bicontinuous. This is because it has been shown that, with an increase in water content, the conductivity of W/O microemulsions incorporating nonionic surfactants increases, while in the case of bicontinuous emulsions, it varies very little, and for O/W systems, it decreases [72,77]. Moreover, a sudden and pronounced increase of conductivity in the domain with low water content corresponding to W/O microemulsions was shown to be indicative of a water percolation phase transition [74,75]. Up to the water percentage corresponding to this sudden increase, inverse micelles are scattered in the oil matrix, but at the water percolation point, they approach each other enough to form clusters, whose number increases from this point onward, and implicitly, there is an increase in electric conductivity. A further increase of water content leads to the appearance of bicontinuous domains (intertwined domains of water and oil) and then, when there is sufficient water, phase inversion occurs and the microemulsion becomes O/W, with oil-based normal micelles dispersed into water. Any additional water added past the phase inversion point leads only to the dilution of these micelles and thus a decrease in overall conductivity.

**Figure 12:**
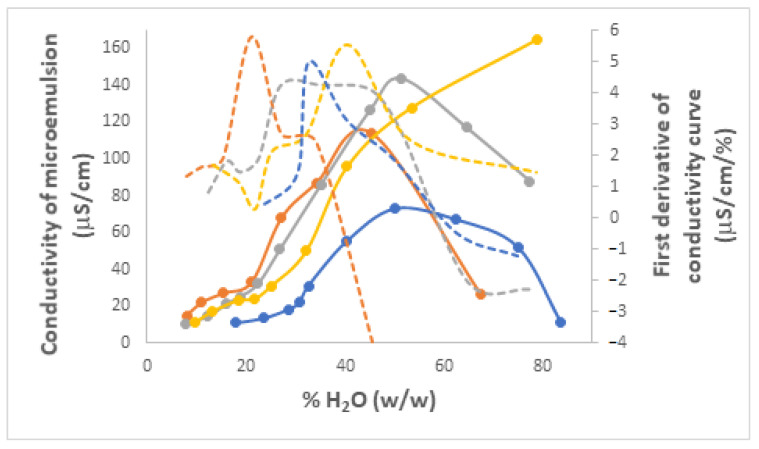
Electrical conductivity variation curves (full) and their first order derivatives (dashed) as a function of the weight % of H_2_O incorporated for Winsor IV microemulsion systems H_2_O/Brij 30/PRAC without and with different proportions of added C3-ol co-surfactant. R is the volumetric ratio H_2_O/C3-ol, color coded as follows: R 2 (orange), R 3 (grey), R 4 (yellow), Without C3-ol (blue). t = 25 °C.

The curves that depict the variation of conductivity for the same selection of microemulsions as a function of their water weight percent are shown in Figure 12 (PRAC) and Figure S3 (ETPR), overlaid with their first-order derivative curves that reveal better local maxima corresponding to sudden conductivity changes at percentages of water where the percolation transition occurs in each system [75]. All figures generally show the expected behavior, with slight conductivity increases at first in the W/O domain, then a sudden increase up to a maximum, followed usually by a decrease along the O/W domain. The only exception is the systems with a water/isopropanol R ratio of 4 (Figure 12 and Figure S3) where only the water percolation transition is observed, but not the decrease following the phase inversion, which is likely the result of the limited range of water percentages explored, from 0 up to only 80%. Overall, there is a shift towards lower water percentages of both percolation transition and phase inversion points as the C3-ol content increases (lower R values), which suggests that the alcohol co-surfactant facilitates the joining and then the merger of water molecules.

Given the nonionic nature of the surfactant, it comes as no surprise that conductivity values remained very low in all cases, from about 5 to 160 µS/cm. The conductivity of microemulsions incorporating only nonionic species is thought to be the outcome of statistical equilibrium fluctuations of micelle charges around a zero mean, where pairs of initially neutral, neighboring micelles approach each other and undergo disproportionation leading to oppositely charged micelles: 2 M → M^+^ + M^−^ [78,79]. For microemulsions composed of nonionic EL-35 (poloxyl 35), water, vitamin E and ethyl butyrate as oil, and ethanol, Feng et al. measured conductivities from about 10 to 160 µS/cm, very similar to ours [72]. Working with microemulsions made of Tween 20, water, benzene, and various alcohol co-surfactants, Mehta and Bala also obtained similar conductivities, from ~1 to 150 µS/cm [75]. Xu et al. measured conductivities in the range of 5–200 µS/cm for microemulsions made of water, Tween 20, and algal oil and observed the same increasing trend in the W/O area followed by a decrease in the O/W domain [77].

#### 3.3.4. Viscosities of Microemulsions

The viscosity of microemulsions is not only a very important physical and transport property, reflecting their resistance to flow, but also another parameter that can be used in practice to distinguish between W/O and O/W microemulsions since the increase of water content leads to an increase in viscosity for the former and a decrease for the latter [72]. Furthermore, should a bimodal profile of viscosity as a function of increasing water content be obtained, i.e., two local maxima and one local minimum in between, this is typically assigned to a phase inversion phenomenon [74,75].

**Figure 13:**
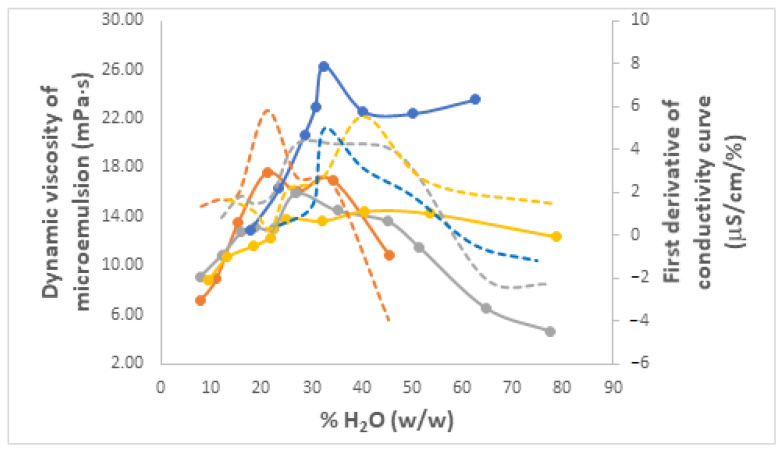
Dynamic viscosity variation curves (full) and the first order derivatives of conductivity curves (dashed) as a function of the weight % of H_2_O incorporated for Winsor IV microemulsion systems H_2_O/Brij 30/PRAC without and with different proportions of added C3-ol co-surfactant. R is the volumetric ratio H_2_O/C3-ol, color coded as follows: R 2 (orange), R 3 (grey), R 4 (yellow), Without C3-ol (blue). t = 25 °C.

The dynamic viscosities of the same selection of Winsor IV microemulsions, based on either PRAC or ETPR as an ester component, Brij 30 as a surfactant, and either water or a mixture of water and C3-ol with a volumetric ratio R, were measured at 25 °C with an automated microviscometer AMVn (Anton Paar), which relies on the rolling-ball method of measurement. The curves showing the change of viscosities with increasing water content are shown in Figure 13 and Figure S4 for PRAC and ETPR, respectively, overlaid with the first-order derivative conductivity curves from Section 3.3.3. In almost all cases, viscosity profiles are indeed bimodal. Apparent exceptions are the two microemulsions lacking a co-surfactant (Figure 13 and Figure S4), where the second maximum is not fully developed because viscosity measurements were not possible past the 60 or 70% H_2_O limits shown as a consequence of gel formation. A closer look reveals that, for all samples, their two maxima in viscosity are perfectly aligned with or at least very close to the local maxima of the matching conductivity derivative curves, hence they correspond to sudden conductivity changes (Figure 13 and Figure S4). Thus, the first viscosity maximum marks the water percolation transition within the W/O microemulsion existence domain. It is due to the formation of an aqueous microdomain within the ester dispersion medium, resulting from the build-up of inverse micelle clusters that precedes the bicontinuous structure [75]. The second viscosity maximum appears within the bicontinuous domain, just before phase inversion happens, which is why viscosity drops sharply beyond this second maximum, a phenomenon that is a characteristic of O/W microemulsions and is the result of the dilution of normal micelles dispersed in the continuous water environment.

As mentioned before, one of the acclaimed advantages of microemulsions is their low viscosity. For the systems studied herein, this ranged from a low of 2 to a high of 30 mPa·s. Similar values were reported for other related systems. For example, Mehta and Bala reported dynamic viscosities from about 2 to 50 mPa·s for their systems with Tween 20, benzene, water, and several alcohol co-surfactants [75]. Working with a more complex composition of Miglyol 812N oil (mostly mid-chain glycerides), water, Solutol HS 15, and Span 80 mixed nonionic surfactants and ethanol co-surfactant, Ren et al. also observed Newtonian behavior for their microemulsions, but with somewhat higher viscosities from 57 to 92 mPa·s at 20 °C, but still below 100 mPa·s [80].

#### 3.3.5. Micellar Particle Sizes and Polydispersity Indices (PDIs)

Hydrodynamic diameters and PDIs were determined for the micelles that make up a selection of Winsor IV microemulsions with a variety of initial water-to-oil volumetric ratios (*V_Water_*/*V_Ester_*), but with water always in larger amounts, as well as without and with added C3-ol in the three relative ratios to water mentioned above. The results are shown in Table 5 for PRAC-based emulsions and Table S3 for the ETPR-based ones.

There is some size variability noticeable in the two tables. Based on the conductivity and viscosity data presented in the sections above, there is an indication that phase inversion tends to occur around a ratio *V_Water_*/*V_Ester_* of around 5.7 for the majority of the systems. The only exceptions are the two pseudo-ternary systems with R = 4 where phase inversion happens at a ratio *V_Water_*/*V_Ester_* of 19 or above as they have not shown conductivity maxima within the limited range of compositions plotted and the microemulsion with PRAC devoid of C3-ol where the phase inversion takes place around a *V_Water_*/*V_Ester_* of 3. In this respect, it is noticeable in the data that larger variations in size happen when there is a change in the nature of micelle particles, from inverse micelles to normal micelles, i.e., when the microemulsion inverts from W/O to O/W. In the cases where the system also contains the co-surfactant C3-ol, the normal micelles have larger diameters (>50 nm) while inverse micelles are smaller (<50 nm). For W/O microemulsions, as water content increased, surfactant content decreased as there was lesser demand for it for the formation of Winsor IV microemulsions (see Section 3.1). Consequently, with few exceptions, inverse micelles grow upwards in Table 5 and Table S3 as there is less and less surfactant available, which needs to enclose more and more water. Inverse micelles are known to be a lot more prone to the phenomenon of coalescence than normal micelles [81]. After the phase inversion happens, the resulting normal micelles could be either larger or smaller than the maximum size reached by corresponding inverse micelles just before their inversion. The systems with a 19-fold excess of water in comparison to oil formed gels rather than liquid emulsions at room temperature; therefore, their particle sizes could not be measured. PDI values remained below 0.7 in all instances, which is indicative of a low spread of particle dimensions [82].

### 3.4. Application of Ester-Based Microemulsions for the Extraction of Tricyclic Antidepressant Drugs from Aqueous Media

#### 3.4.1. Comparison of Extraction Efficiencies into Winsor II Microemulsions Using Seven Different Homologous Ester Oils

Winsor II microemulsions using seven of the nine ester homologs (excluded were the highly volatile and thus inconvenient MEAC and ETFO) were investigated as potential extraction solvents for tricyclic antidepressants (TCA) from aqueous solutions, i.e., laboratory-simulated wastewater contaminated with such drug residues. Antidepressants were chosen as model compounds for water contaminants as they represent one pharmaceutical class that has witnessed a boom lately in prescription and consumption rates worldwide [83,84,85] and, therefore, their residues found in wastewater are of increasing environmental concern, especially considering their reported negative effects on aquatic life [86].

**Figure 14:**
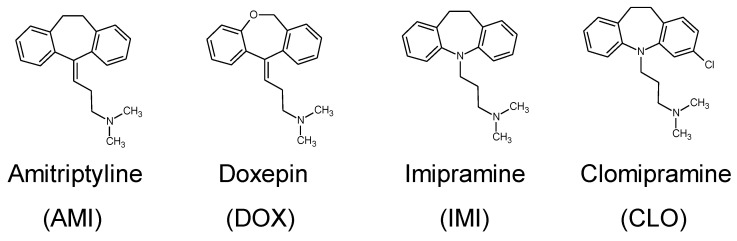
Chemical structures of the four tricyclic antidepressants used as model pharmaceutical contaminants of water in this work.

All extractions were performed starting with 9 mL of TCA aqueous solution to which 1 mL of ester was added (to ensure a sufficiently large excess for recovery of purified water after phase separation at equilibrium) and then the amount of Brij 30 necessary to form a Winsor II microemulsion (according to the phase diagrams determined in Section 3.1) was added drop-wise under gentle stirring, i.e., the extracting microemulsion was generated in situ. The four TCAs investigated in this work are hydrochlorides of organic tertiary amines (Figure 14); therefore, they tend to be more hydrophilic than hydrophobic and have a greater preference for water than ester oil.

**Figure 15:**
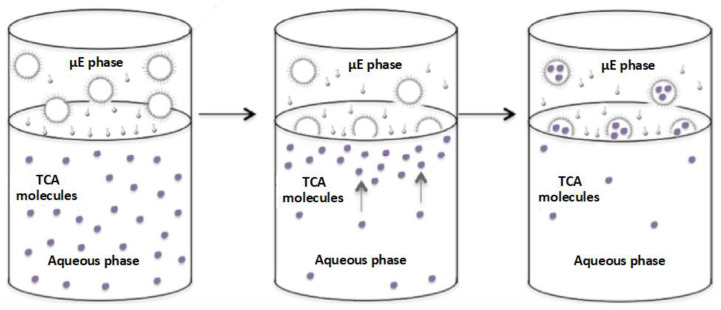
Schematic of the principle of extraction of TCA hydrophilic contaminants from the aqueous solution into the microemulsion (µE) upper phase. Filled small circles represent the TCA molecules, the tailed dots are surfactant molecules, and the larger circles with attached tailed dots around their circumference represent the inverse water-containing micelles (adapted from [28]).

A diagram depicting the principle of extraction of TCAs from the aqueous medium into the inverse micelles of the Winsor II microemulsion is shown in Figure 15. TCA molecules dissolved in water diffuse towards the interface with the organic (microemulsion) layer where they are incorporated into the aqueous micelles coated with a surfactant shell that disperses them into the ester oil medium until equilibrium is reached.

Table 6 shows the extraction yields (η) from water into the microemulsion (µE) and Nernst distribution coefficients (*K_D_*) between microemulsion and water achieved for the four TCAs (each with an initial concentration of 20 mg/L) using the seven ester-based microemulsions. An additional relevant parameter for appreciating the extraction performance, which was also included in Table 6, is the water per microemulsion (W/µE) volumetric ratio after equilibration. This is important because it reflects the amount of purified water recovered from the original 9 mL; therefore, a higher ratio is desirable. Although the starting volumetric ratio between water and ester was 9 in all cases, the formation of Winsor II phases in these systems demands very different amounts of Brij 30 as shown in Section 3.1, which implies very different degrees of water incorporation into the Winsor II microemulsions and thus variable recovery of non-emulsified water.

Although the W/µE ratio is highest in the case of ETAC, extraction yields and Nernst coefficients are the worst for this choice of ester, irrespective of the TCA (Table 6). The longer chain esters, namely PEAC, HEAC, and ETBU, lead to higher extraction yields of TCAs, but produce too much microemulsion and thus leave behind too little purified water. In fact, in these cases, the high extraction yields, representing the % of the initial mass of TCA that passed into the microemulsion phase, are due in great part to the fact that there is a much higher volume of microemulsion taking up the TCAs than in the case of other esters. In this respect, *K_D_* would be a better measure of extraction efficiency than η, as it quantifies the amount of TCA per unit volume of µE divided by the amount of TCA per unit volume of W, so equal volumes of the phases in equilibrium are considered in its calculation. Among the remaining esters, ETPR leads to a higher volume of purified water, but at the cost of consistently lower extraction yields and *K_D_* values for all TCAs. This leaves PRAC and BUAC, which both produce almost three times more purified water than microemulsion at equilibrium, but with better yields and *K_D_* coefficients in the case of PRAC. For these reasons, combined with its low volatility and the observed lower consumption of Brij 30 when preparing its Winsor II systems (see Section 3.1.1), PRAC was the ester component of choice for the preparation of Winsor II microemulsions in all extraction experiments to follow.

#### 3.4.2. Determination of Extraction Yields and Distribution Coefficients into PRAC-Based Microemulsions for Different Initial Loadings of Water with TCAs at pH 6

The variability of the extraction efficiency of the PRAC emulsion with an initial H_2_O/PRAC volumetric ratio equal to 9 from several aqueous solutions with different concentrations of TCAs was also tested (Table 7). The pH of all aqueous TCA solutions was measured and found to be around 6, while the range of initial TCA concentrations was from 5 to 100 mg/L, which are rather large concentrations compared to the typical concentrations of pharmaceutical emerging contaminants found in water streams, in the order of ppb (µg/L) [87]. While there were slight drops in extraction yields with increasing initial concentrations for all TCAs, even at the largest initial loading level, extraction yields remained around 90% and there was no indication of saturation of microemulsions with any of the TCA contaminants within our working range (Table 7). While it cannot be ruled out that at some higher contaminant loading level of the water, there may occur a saturation point past which the microemulsion micelles can no longer incorporate any more TCAs, this saturation level is likely very high compared to the realistic concentrations of such contaminants in wastewater.

**Figure 16:**
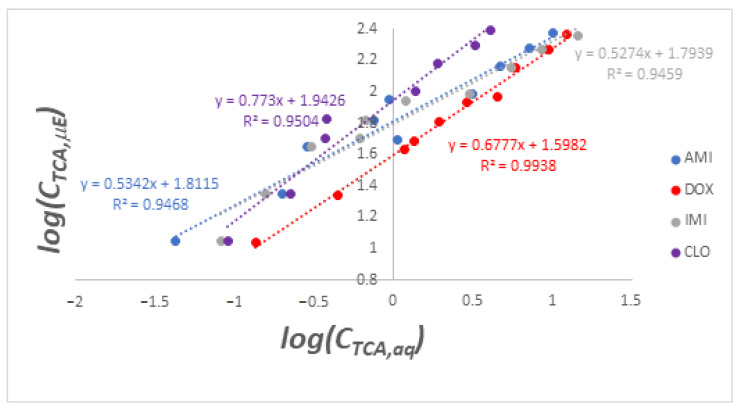
Logarithmic plots of concentrations of TCAs extracted into microemulsions against concentrations remaining in the aqueous phase.

The distribution coefficient *K_D_* of a given TCA represents the ratio between the concentration of that TCA in the microemulsion (*C_TCA,µE_*) and its concentration in the aqueous phase (*C_TCA,aq_*) after equilibrium is reached between the two phases. Rather than calculating this ratio for every data point, a more accurate way of determining *K_D_* over the whole data set corresponding to each TCA is to plot the $\log\left(C_{TCA,\mu E}\right)$ against the $\log\left(C_{TCA,\mu E}\right)$ and obtain the $\log\left(K_{D}\right)$ value as the y-intercept of the resulting linear plot [50]. Figure 16 shows the linear plots corresponding to the four TCAs and Table 8 is a centralization of *K_D_* values obtained for each of these.

The data in Table 8 show that all distribution coefficients are in order of a few tens, clearly favoring the partitioning of TCAs into the microemulsion as opposed to water. A somewhat higher preference for the microemulsion for CLO and a somewhat lower one for DOX are also apparent, while AMI and IMI seem to be equally well extracted. These trends are also noticeable upon a closer inspection of the data in Table 7 as well. Unlike the situation in Section 3.3.1, this time extraction yields are an accurate reflection of extraction performance because the same type of PRAC-based microemulsion is used as an extraction medium for all TCAs and all concentrations, with a consistent W/µE ratio of 2.7.

As ionic compounds, the limiting solubilities of TCA hydrochlorides in water are known to be very high, around 500 mg/mL at ambient temperature [88,89]. Thus, it is somewhat surprising that they show such higher preference (ca. 40- to 88-fold) for the microemulsion phase to the detriment of the water phase. It is the surfactant that likely plays a key role in this sense. The Winsor II microemulsion is of the W/O type, meaning that the surfactant molecules form inverse micelles, with their hydrophobic tails pointing outwards and the more polar polyoxyethylene groups inwards of the micelles, where the TCA hydrochloride molecules are sequestered within an aqueous droplet. Thus, in addition to interactions with water molecules that exist in any aqueous TCA solution, e.g., ion-dipole and hydrogen bonds, other stabilizing intermolecular forces come into play that involve the surfactant coating of the micelle. The oxygen atoms of polyoxyethylene groups can act as additional hydrogen bond acceptors for the acidic protons of TCA hydrochlorides, thus favoring their encapsulation into the micelles of the microemulsion phase as opposed to their spread into the water continuum of the aqueous phase.

#### 3.4.3. Determination of Extraction Yields into PRAC-Based Microemulsions from Aqueous Solutions of TCAs at pH 9 and at High Ionic Strength

Because TCAs are tertiary amines, with pK_a_ values around 9 [90], they are involved in a proton exchange equilibrium in aqueous media:


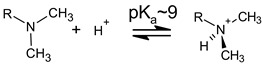


As a consequence of this equilibrium, pH is expected to have a significant effect on the relative proportions of the protonated and non-protonated forms, which have very different affinities for water, the former being ionic and a lot more hydrophilic. It follows that an increase of the pH above 6 (the typical pH value of all aqueous TCA hydrochloride solutions) would cause a shift of the chemical equilibrium in favor of the non-protonated form, which is less hydrophilic and, thus, less soluble in water. This is expected to increase even further the extraction yield of TCAs from water into the W/O microemulsion.

Another factor that may increase extraction yields is the increase of the ionic strength of the aqueous phase through the addition of kosmotropic salts like NaCl. Such salts are known to increase surface tension, enhance the hydrophobic effect, and produce a “salting out” of the aqueous phase of otherwise water-soluble compounds during liquid-liquid partitioning [91,92]. They also increase the density of the water layer easing the phase separation of the microemulsion at the top.

To test these hypotheses, we prepared 100 mg/L TCA solutions using 0.5 M NaCl and phosphate buffer with a pH of 9 as solvents instead of distilled water and repeated the extraction experiments with in situ generated PRAC microemulsions. This specific concentration of NaCl and particular pH value were chosen because they were shown previously to not affect Winsor II phase formation much (Section 3.2.2 and Section 3.2.3). Table 9 presents the extraction yields obtained.

As expected, the already large extraction yields reported in Table 7 were enhanced even further by both the adjustment of pH from acidic to basic and, in general, also by the addition of salt, the only exception being CLO where the yield was slightly smaller (Table 9). In particular, the pH increase led to extraction yields of almost 100% in all cases, which translates into the complete removal of pharmaceutical contaminants from water. There is a possibility that this excellent outcome is actually the combined result of both the basic pH and salting out effect by buffer ions, as phosphate anions are also known to be kosmotropic [93].

These results suggest that both pH and ionic strength adjustments of wastewater containing antidepressant contaminants could be included in wastewater treatment procedures prior to microemulsion-based extraction to enhance decontamination efficiency.

## 4. Conclusions

Winsor II and IV microemulsion systems were fabricated successfully from water and all nine homologous esters investigated in this work using titration with Brij 30, PRAC, and ETPR being the esters with the largest microemulsion domains and the smallest surfactant consumptions. The addition of a co-surfactant, isopropanol, often increased surfactant consumption for system compositions high in water, so it presented no significant advantage for the extraction of analytes from aqueous media. The presence of NaCl in the aqueous phase increased surfactant consumption for obtaining Winsor IV microemulsions, but had little effect on Winsor II formation when the concentrations of NaCl remained below 1 M. An acidic pH in the aqueous phase increased the Brij 30 consumption needed to reach Winsor II and IV microemulsions, but basic pHs did not affect this much. Microemulsion densities of Winsor IV systems appeared to be influenced primarily by their water content, while refractive indices were influenced by the surfactant content. Conductivity and viscosity measurements helped delineate W/O from O/W microemulsions. The addition of isopropanol as a co-surfactant shifted the phase inversion points to lower water contents in comparison to ternary systems lacking the co-surfactant. Micelle sizes remained below 100 nm both with and without the co-surfactant.

PRAC was also the best choice of ester for fabricating Winsor II microemulsions for the purpose of extracting tricyclic antidepressant residues from water, leading to the best extraction yields, highest Nernst distribution coefficient, and a good ratio of recovered water per microemulsion produced. Increasing the ionic strength of the aqueous phase or shifting the pH to more basic values were shown to have a beneficial effect on the extraction of TCA drugs into PRAC-based microemulsions.

With the exception of ETAC and BUAC systems, which have been described before, all the other Brij 30- and ester-based systems in this paper are reported here for the first time. This is also the first report about the successful application of in situ-prepared microemulsions for the extraction of pharmaceutical residues contaminating the aqueous environment. Aside from the fundamental insights into microemulsion phase behavior provided by our studies and the particular practical application evidenced in this work, considering the structural similarity between TCAs and many other drug classes, these findings recommend ester and nonionic surfactant aqueous microemulsions as extraction systems for the depollution of water contaminated with a wide range of pharmaceuticals and personal care products.

## Figures and Tables

**Table 1:** Relative areas (% of total area) of domains delimited on the ternary diagrams of systems incorporating water, Brij 30, and homologous alkyl acetate esters.

Ester C_x_ + C_2_ (x = 1–6) Incorporated in the Microemulsion	% Area of Domain Found under the Winsor II Curve	% Area of Winsor II Microemulsion Domain	% Area of Winsor IV Microemulsion Domain
MEAC (x = 1)	12.97	11.46	52.48
ETAC (x = 2)	17.87	12.52	42.26
PRAC (x = 3)	15.44	10.84	47.10
BUAC (x = 4)	21.65	23.93	44.46
PEAC (x = 5)	18.46	17.70	26.06
HEAC (x = 6)	24.71	12.60	26.07

**Table 2:** Relative areas (% of total area) of domains delimited on the ternary diagrams of systems incorporating water, Brij 30, and homologous ethyl carboxylate esters.

Ester C_2_ + C_y_ (y = 1–4) Incorporated in the Microemulsion	% Area of Domain Found under the Winsor II Curve	% Area of Winsor II Microemulsion Domain	% Area of Winsor IV Microemulsion Domain
ETFO (y = 1)	17.73	6.62	47.75
ETAC (y = 2)	17.87	12.52	42.26
ETPR (y = 3)	14.81	10.12	44.63
ETBU (y = 4)	34.65	6.70	25.63

**Table 3:** Relative areas (% of total area) of domains delimited on the pseudo-ternary diagrams of systems incorporating water/C3-ol in a volumetric ratio R, Brij 30, and PRAC.

H_2_O/C3-ol Volumetric Ratio R	% Area of Domain Found under the Winsor II Curve	% Area of Winsor II Microemulsion Domain	% Area of Winsor IV Microemulsion Domain
R = 2	22.79	4.93	33.48
R = 3	16.13	14.32	32.87
R = 4	19.98	22.16	38.67
Without C3-ol	15.44	10.84	47.10

**Table 4:** Relative areas (% of total area) of domains delimited on the pseudo-ternary diagrams of systems incorporating water/C3-ol in a volumetric ratio R, Brij 30, and ETPR.

H_2_O/C3-ol Volumetric Ratio R	% Area of Domain Found under the Winsor II Curve	% Area of Winsor II Microemulsion Domain	% Area of Winsor IV Microemulsion Domain
R = 2	17.02	20.28	36.97
R = 3	27.68	22.63	33.88
R = 4	12.88	39.48	29.45
Without C3-ol	14.81	10.12	44.63

**Table 5:** Particle diameters and polydispersity indices (PDIs) for micelles of Winsor IV microemulsions prepared with PRAC as ester oil, water, Brij 30, without and with isopropanol (C3-ol) added as a co-surfactant. R represents the volumetric ratio between water and C3-ol. The initial volumetric ratio between water and ester (*V_Water_*/*V_Ester_*) is also given in the first column. Results are reported as average ± standard deviation (n = 3).

$\frac{V_{Water}}{V_{Ester}}$	Particle Diameter, nm	PDI	Particle Diameter, nm	PDI	Particle Diameter, nm	PDI	Particle Diameter, nm	PDI
	Without C3-ol		R = 2		R = 3		R = 4	
19.0	gel		74.66 ± 1.52	0.20 ± 0.05	54.30 ± 0.37	0.14 ± 0.04	44.45 ± 1.66	0.13 ± 0.03
5.7	85.17 ± 8.70	0.57 ± 0.05	32.89 ± 0.18	0.11 ± 0.01	26.25 ± 0.37	0.16 ± 0.02	37.82 ± 1.03	0.20 ± 0.04
3.0	63.31 ± 0.88	0.17 ± 0.08	22.03 ± 0.08	0.23 ± 0.03	20.86 ± 0.13	0.21 ± 0.01	34.05 ± 1.24	0.22 ± 0.02
1.9	47.40 ± 3.67	0.19 ± 0.01	21.12 ± 1.32	0.30 ± 0.04	33.65 ± 1.88	0.21 ± 0.04	24.83 ± 0.86	0.24 ± 0.03

**Table 6:** Water over microemulsion volumetric ratios (W/µE), extraction yields (η), and distribution coefficients (*K_D_*) were obtained when extracting 20 mg/L AMI, DOX, IMI, and CLO from water into microemulsions based on seven homologous esters as oil component and Brij 30 as a surfactant (t = 25 °C).

Ester	W/µE Ratio	AMI 20 mg/L		DOX 20 mg/L		IMI 20 mg/L		CLO 20 mg/L	
		η (%)	*K_D_*	η (%)	*K_D_*	η (%)	*K_D_*	η (%)	*K_D_*
ETAC	4.8	67.2	9.9	35.5	3.3	77.1	14.0	82.5	28.3
PRAC	2.7	95.5	63.8	94.4	39.3	97.4	60.9	98.4	87.8
BUAC	2.7	90.6	25.7	71.1	6.6	85.0	15.1	97.2	92.3
PEAC	1.9	95.6	41.2	65.2	4.3	91.0	19.0	95.2	45.8
HEAC	0.7	99.0	67.1	97.7	30.2	98.5	43.6	99.5	166.9
ETPR	3.4	84.0	18.2	62.7	5.7	79.7	13.4	91.2	35.2
ETBU	0.8	98.7	59.7	96.3	23.8	98.4	40.6	99.3	132.5

**Table 7:** Extraction yields of TCAs into H_2_O/Brij 30/PRAC microemulsion as a function of initial concentrations of TCAs in water (t = 25 °C; pH = 6).

TCA	Initial Concentration in Water, mg/L	Extraction Yield η, %
AMI	5	99.3
	10	98.4
	20	95.5
	30	98.0
	40	93.4
	60	93.5
	80	92.4
	100	91.5
DOX	5	97.9
	10	96.5
	20	94.4
	30	94.9
	40	90.5
	60	91.8
	80	90.1
	100	89.7
IMI	5	98.7
	10	98.8
	20	97.4
	30	98.3
	40	93.7
	60	92.3
	80	90.9
	100	88.0
CLO	5	98.6
	10	98.2
	20	98.4
	30	97.1
	40	97.1
	60	97.4
	80	96.5
	100	96.6

**Table 8:** Distribution coefficients of TCAs between the H_2_O/Brij 30/PRAC microemulsion and water (t = 25 °C; pH = 6).

TCA	log*K_D_*	*K_D_*
AMI	1.812	64.8
DOX	1.598	39.6
IMI	1.794	62.2
CLO	1.943	87.6

**Table 9:** Extraction yields of TCAs into an H_2_O/Brij 30/PRAC microemulsion when the aqueous phase has high ionic strength (0.5 M NaCl) or a pH of 9 (phosphate buffer). t = 25 °C.

TCA and Concentration	η (%) aq. NaCl 0.5 M	η (%) aq. pH 9
AMI 100 mg/L	99.06	99.95
DOX 100 mg/L	98.88	99.97
IMI 100 mg/L	99.80	99.96
CLO 100 mg/L	93.61	99.93

## Data Availability

The data are available on request from the corresponding authors.

## Supplementary Materials

The following supporting information can be downloaded at: https://www.mdpi.com/article/10.3390/nano13162311/s1. Figure S1: Variation of densities as a function of the weight % of H_2_O incorporated for Winsor IV microemulsion systems H_2_O/Brij 30/ETPR without and with different proportions of added C3-ol co-surfactant; Figure S2: Variation of refractive indices as a function of the weight % of H_2_O incorporated for Winsor IV microemulsion systems H_2_O/Brij 30/ETPR without and with different proportions of added C3-ol co-surfactant; Figure S3: Electrical conductivity variation curves and their first order derivatives as a function of the weight % of H_2_O incorporated for Winsor IV microemulsion systems H_2_O/Brij 30/ETPR without and with different proportions of added C3-ol co-surfactant; Figure S4: Dynamic viscosity variation curves and the first order derivatives of conductivity curves as a function of the weight % of H_2_O incorporated for Winsor IV microemulsion systems H_2_O/Brij 30/ETPR without and with different proportions of added C3-ol co-surfactant; Table S1: PRAC-based Winsor IV microemulsion densities calculated based on a weighted average of the densities of pure components, with mass percentages taken as weights, experimental densities, and relative % deviations of experimental densities from the calculated ones; Table S2: ETPR-based Winsor IV microemulsion densities calculated based on a weighted average of the densities of pure components, with mass percentages taken as weights, experimental densities, and relative % deviations of experimental densities from the calculated ones; Table S3: Particle diameters and polydispersity indices (PDIs) for micelles of Winsor IV microemulsions prepared with ETPR as ester oil, water, Brij 30, without and with isopropanol (C3-ol) added as co-surfactant.
