# Supplementary material for: Microemulsions of Nonionic Surfactant with Water and Various Homologous Esters: Preparation, Phase Transitions, Physical Property Measurements, and Application for Extraction of Tricyclic Antidepressant Drugs from Aqueous Media
Source: Nanomaterials (Basel). 2023 Aug 11;13(16):2311. doi: 10.3390/nano13162311 (PMC10458553; doi:10.3390/nano13162311)
Supplement: Supplementary file 1 [file nanomaterials-13-02311-s001.zip › nanomaterials-2560473-supplementary.pdf]

Supplementary Information

Microemulsions of Nonionic Surfactant with Water and Various Homologous Esters: Preparation, Phase Transitions, Physical Property Measurements, and Application for Extraction of Tricyclic Antidepressant Drugs from Aqueous Media

Radu C. Racovita [1,*], Maria D. Ciuca [1], Daniela Catana [1], Cezar Comanescu [1,2,3,*] and Oana Ciocirlan [1]

[1] Department of Inorganic Chemistry, Physical Chemistry and Electrochemistry, Faculty of Chemical Engineering and Biotechnologies, National University of Science and Technology Politehnica Bucharest, 1-7 Gh. Polizu St., District 1, 011061 Bucharest, Romania; maria_daniela.ciuca@upb.ro (M.D.C.); daniela.catana2911@upb.ro (D.C.); oana.ciocirlan@upb.ro (O.C.)
[2] National Institute of Materials Physics, 405A Atomistilor St., 077125 Magurele, Romania
[3] Faculty of Physics, University of Bucharest, 405 Atomistilor St., 077125 Magurele, Romania
* Correspondence: radu.racovita@upb.ro (R.C.R.); cezar.comanescu@infim.ro (C.C.)

Supplementary Figures:

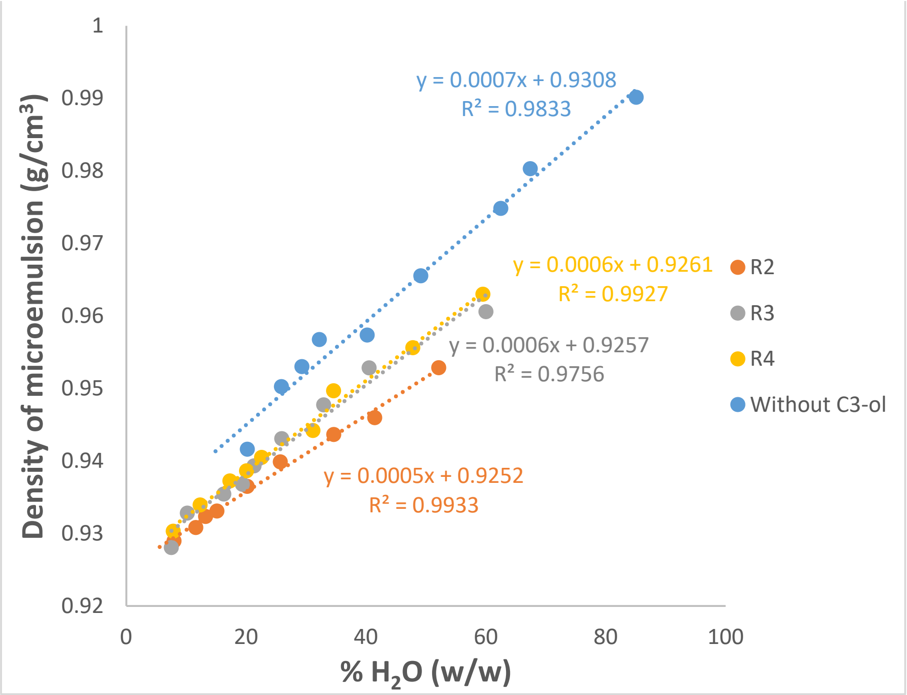

Figure S1. Variation of densities as a function of the weight % of H_2O incorporated for Winsor IV microemulsion systems H_2O/Brij 30/ETPR without and with different proportions of added C3-ol co-surfactant. R is the volumetric ratio H_2O/C3-ol. t=25^{0}C.

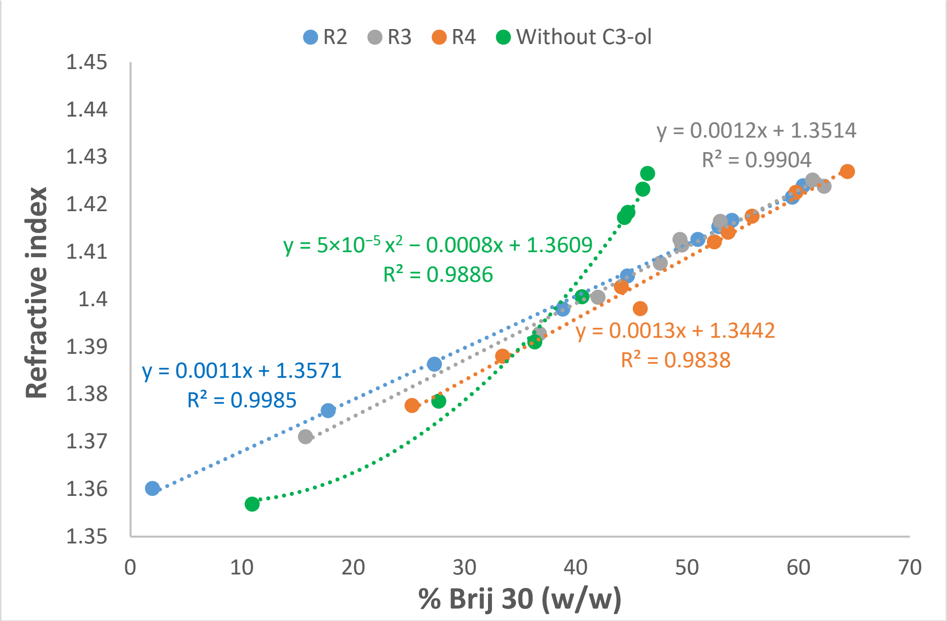

Figure S2. Variation of refractive indices as a function of the weight % of H_2O incorporated for Winsor IV microemulsion systems H_2O/Brij 30/ETPR without and with different proportions of added C3-ol co-surfactant. R is the volumetric ratio H_2O/C3-ol. t=25^{0}C.

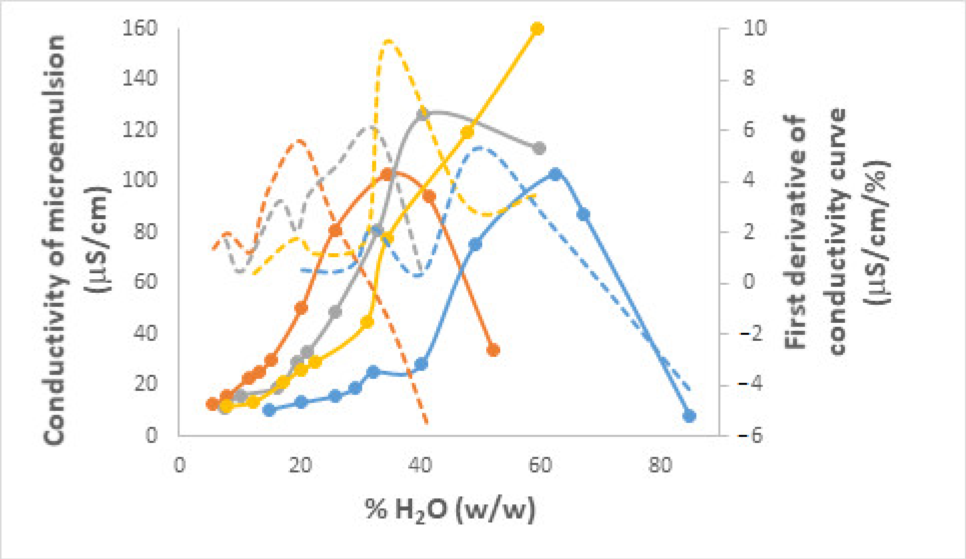

Figure S3. Electrical conductivity variation curves (full) and their first order derivatives (dashed) as a function of the weight % of H_2O incorporated for Winsor IV microemulsion systems H_2O/Brij 30/ETPR without and with different proportions of added C3-ol co-surfactant. R is the volumetric ratio H_2O/C3-ol, color coded as follows: R 2 (orange), R 3 (grey), R 4 (yellow), Without C3-ol (blue). t=25^{0}C.

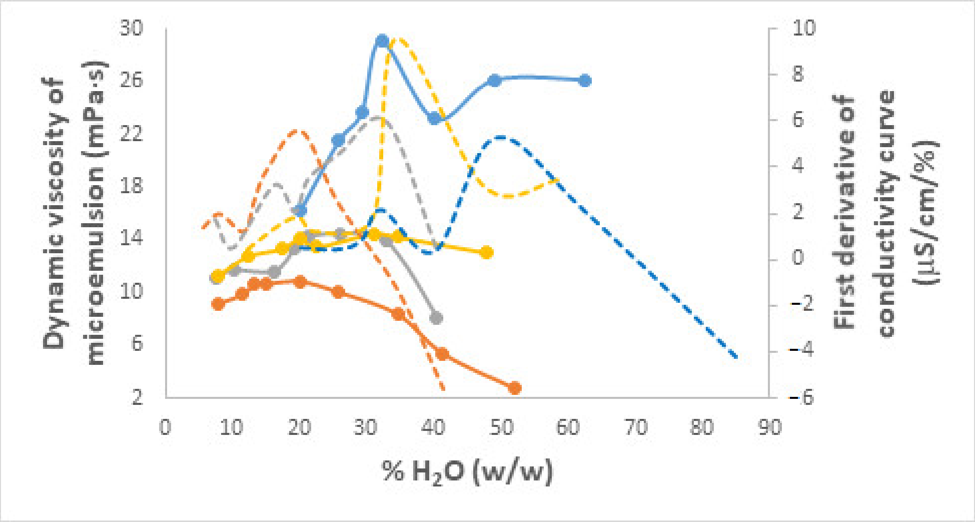

Figure S4. Dynamic viscosity variation curves (full) and the first order derivatives of conductivity curves (dashed) as a function of the weight % of H_2O incorporated for Winsor IV microemulsion systems H_2O/Brij 30/ETPR without and with different proportions of added C3-ol co-surfactant. R is the volumetric ratio H_2O/C3-ol, color coded as follows: R 2 (orange), R 3 (grey), R 4 (yellow), Without C3-ol (blue). t=25^{0}C.

Supplementary Tables:

Table S1. PRAC-based Winsor IV microemulsion densities calculated based on a weighted average of the densities of pure components, with mass percentages taken as weights, experimental densities, and relative % deviations of experimental densities from the calculated ones. R represents the volumetric ratio between water and co-surfactant C3-ol in the case of four-component systems.

Sample no.	Calculated density, g/cm³	Measured density, g/cm³	Dev., %	Calculated density, g/cm³	Measured density, g/cm³	Dev., %	Calculated density, g/cm³	Measured density, g/cm³	Dev., %	Calculated density, g/cm³	Measured density, g/cm³	Dev., %
	Without C3-ol			*R = 2*			*R = 3*			*R = 4*		
1	0.98686	0.98957	0.275	0.93532	0.95403	2.000	0.95002	0.96122	1.179	0.95869	0.96394	0.547
2	0.98057	0.98304	0.252	0.93445	0.94596	1.232	0.94778	0.95664	0.935	0.95128	0.95597	0.493
3	0.97323	0.97613	0.298	0.93300	0.94239	1.006	0.94585	0.95368	0.828	0.94653	0.95063	0.433
4	0.96420	0.96521	0.104	0.93182	0.93715	0.572	0.94267	0.9485	0.618	0.94263	0.94485	0.235
5	0.95680	0.95737	0.059	0.92940	0.93455	0.555	0.94029	0.94443	0.440	0.93935	0.94064	0.137
6	0.95042	0.95551	0.536	0.92848	0.92866	0.019	0.93725	0.93955	0.245	0.93798	0.93838	0.043
7	0.94727	0.95219	0.520	0.92869	0.92558	-0.335	0.93683	0.93808	0.133	0.93703	0.93653	-0.053
8	0.94391	0.94909	0.548	-	-	-	0.93611	0.93645	0.036	0.93481	0.93324	-0.168
9	0.93663	0.94333	0.716	-	-	-	0.93361	0.93317	-0.048	0.93137	0.92955	-0.196
10	0.92852	0.93887	1.115	-	-	-	0.93289	0.92942	-0.372	0.95869	0.96394	0.547

Table S2. ETPR-based Winsor IV microemulsion densities calculated based on a weighted average of the densities of pure components, with mass percentages taken as weights, experimental densities, and relative % deviations of experimental densities from the calculated ones. R represents the volumetric ratio between water and co-surfactant C3-ol in the case of four-component systems.

Sample no.	Calculated density, g/cm³	Measured density, g/cm³	Dev., %	Calculated density, g/cm³	Measured density, g/cm³	Dev., %	Calculated density, g/cm³	Measured density, g/cm³	Dev., %	Calculated density, g/cm³	Measured density, g/cm³	Dev., %
	Without C3-ol			*R* = 2			*R* = 3			*R* = 4		
1	0.98749	0.99013	0.268	0.92006	0.95284	3.562	0.96903	0.96058	-0.872	0.96975	0.96297	-0.699
2	0.97750	0.98030	0.286	0.91914	0.94596	2.917	0.96737	0.95282	-1.504	0.96838	0.95561	-1.319
3	0.97322	0.97482	0.164	0.91765	0.94363	2.832	0.96649	0.94773	-1.941	0.96710	0.94966	-1.803
4	0.96395	0.96553	0.164	0.91422	0.93987	2.806	0.96574	0.94307	-2.347	0.96636	0.94418	-2.296
5	0.95680	0.95735	0.057	0.90987	0.93649	2.926	0.96508	0.93932	-2.669	0.96556	0.94049	-2.597
6	0.95041	0.95674	0.666	0.90757	0.93309	2.811	0.96476	0.93676	-2.902	0.96523	0.93862	-2.757
7	0.94740	0.95301	0.593	0.90590	0.93233	2.918	0.96450	0.93542	-3.015	0.96492	0.93723	-2.870
8	0.94448	0.95024	0.609	0.90376	0.93080	2.991	0.96421	0.93279	-3.259	0.96438	0.93392	-3.159
9	0.93852	0.94161	0.330	0.90352	0.92896	2.816	0.96367	0.92804	-3.697	0.96398	0.93030	-3.494

Table S3. Particle diameters and polydispersity indices (PDIs) for micelles of Winsor IV microemulsions prepared with ETPR as ester oil, water, Brij 30, without and with isopropanol (C3-ol) added as co-surfactant. R represent the volumetric ratio between water and C3-ol. The initial volumetric ratio between water and ester (V_{water}/V_{ester}) is also given in the first column. Results are reported as average ± standard deviation (n=3).

$\frac{V_{Water}}{V_{Ester}}$	Particle diameter, nm	PDI	Particle diameter, nm	PDI	Particle diameter, nm	PDI	Particle diameter, nm	PDI
	Without C3-ol		*R* = 2		*R* = 3		*R* = 4	
19.0	gel		79.93±1.18	0.23±0.03	64.45±0.08	0.08±0.08	35.50±0.31	0.09±0.02
5.7	89.1±7.21	0.27±0.02	35.98±0.70	0.18±0.04	23.73±0.22	0.19±0.01	30.44±0.40	0.12±0.04
3.0	61.57±1.04	0.09±0.01	37.27±5.43	0.29±0.08	24.74±1.01	0.27±0.03	27.65±0.51	0.22±0.01
1.9	55.74±6.20	0.19±0.03	31.56±4.38	0.27±0.06	30.73±0.98	0.24±0.05	29.43±0.52	0.29±0.03
